# COVID-19 response and the unhoused communities in Sacramento: a mixed methods study with policy implications

**DOI:** 10.1186/s12889-025-24515-0

**Published:** 2025-11-18

**Authors:** Annica Stull-Lane, Kiré Lau, Monica Keiko Lieng, Raquel Selcer, Jericho Hallare, Andrew Maneval, Tanya Talwar, Kirk Harter, Jason Tang, Ellen Shank, Christina Lowry, Silvia Bastea, Lillian Jundi, Nikita Sanghavi, Karli Matter, Tess Hill, Erika Jane Adams, Corinne Cushing, Nitya Janardhan, Duane Kim, Fransia De Leon, Azaam Mamoor, Michael Wilkes

**Affiliations:** 1 School of Medicine, University of California, Davis, 4610 X Street, Sacramento, CA 95817 USA; 2 College of Medicine, California Northstate University, 9700 West Taron Drive, Elk Grove, CA 95757 USA; 3 Office of the Dean, School of Medicine, University of California, Davis, 4610 X Street, Sacramento, CA 95817 USA

**Keywords:** Homelessness, COVID-19, Disaster response, Mixed methods, Health policy

## Abstract

**Background:**

In an emergency response to the COVID-19 pandemic, cross-sector public health collaborations facilitated emergency hotel room placements for people experiencing homelessness (PEH). To inform a Health-in-All-Policies approach to future disasters, the study objective was to assess the impact of the disaster response on the unhoused communities surrounding Sacramento.

**Methods:**

Our interdisciplinary team-based convergent parallel mixed methods study included quantitative and qualitative data collection and analysis, followed by integrated analyses. The quantitative primary outcome was number of self-reported primary care physician visits. Semi-structured in-person interviews focused on perspectives of the COVID-19 pandemic and public health interventions, experiences with healthcare access and preventative measures, and personal priorities. Interviews were recorded, transcribed, and coded using thematic analysis. Integration was guided by the “following a thread” approach.

**Results:**

We surveyed 100 people living outdoors (“outside PEH”) and 100 people in temporary hotel room placements (“hotel PEH”), and we conducted and transcribed interviews with 19 outside PEH and 16 hotel PEH for qualitative thematic analysis. Hotel PEH reported significantly more primary care physician visits compared to outside PEH (*p* < 0.05). Mobile health clinics were viewed favorably by both outside PEH (93%) and hotel PEH (85%). Four qualitative themes emerged: (1) Access to resources; (2) Social connection as empowerment; (3) Exacerbation of pre-existing conditions; and (4) Impact of systems and policy on safety. Integrated comparison demonstrated that hotel placements improved access to information, healthcare, resources, hygiene, hope, and safety. This informed four disaster response guidelines: (1) Prioritize housing; (2) Maintain safe and healthy living environments; (3) Improve health and access to integrated healthcare services; and (4) Promote social and emotional well-being.

**Conclusions:**

Comprehensive disaster responses require understanding vulnerable populations’ experiences, perspectives, and priorities. Student-led team science addresses complex questions, revealing pandemic response lessons and informing Health-in-All-Policies approaches to ongoing and future disasters.

**Supplementary Information:**

The online version contains supplementary material available at 10.1186/s12889-025-24515-0.

## Background

Our study objective was to assess the impact of the COVID-19 disaster response on the unhoused communities surrounding Sacramento, California, to inform future policies. Early in March 2020, people experiencing homelessness (PEH) were identified as a vulnerable population with increased risk for contracting and spreading the coronavirus disease 2019 (COVID-19) [1–5]. Nationally, PEH have traditionally faced barriers to medical care [6, 7] and a higher prevalence of comorbidities such as hypertension, obesity, diabetes, respiratory disease, and substance use disorders than the general population [8–10]. These factors make this population at higher risk of worse outcomes during infection [11]. Further, providing for this underserved population can be complicated by transience and distrust of the medical system [12]. A public health emergency, such as the COVID-19 pandemic, can exacerbate these vulnerabilities and increase social and economic inequalities, since general guidelines for the public may assume a housed status and overlook the unique challenges of PEH [13]. These complexities make it even more critical for policies to enable a stable living environment, especially during a disaster when access to usual services may be limited [14].

Health-in-All-Policies is an approach that prioritizes health equity by articulating cross-sector collaborations that incorporate considerations of socioeconomic and environmental determinants of health into policymaking [15]. Adverse socioeconomic and environmental conditions can lead to poor health outcomes of PEH [9] and therefore are vital considerations in incorporating comprehensive care for this vulnerable population during the COVID-19 pandemic. Further, since PEH are more likely to have experienced childhood trauma, marginalization and dehumanization in society, comprehensive care also necessitates a trauma-informed approach that optimizes trust and safety [12, 16]. In addition, a “Housing First” approach would focus on shelter as a priority and housing as a human right [17]. A pandemic emergency response runs the risk of focusing solely on health protection from a communicable disease perspective, while side-lining health improvement efforts on preventing epidemics of non-communicable diseases [18]. Policies guiding impactful comprehensive care for PEH would consider all of these factors.

Despite accounting for 12% of the total U.S. population, California is home to nearly half (48%) of the unsheltered PEH in the U.S [26]. In Sacramento County alone, up to 11,000 people experience homelessness at some point during the year [19]. As the pandemic came to California and “shelter-in-place” orders were implemented, hotel rooms across the state were re-purposed to house PEH in the emergency response; these efforts were termed *Project Roomkey* [19, 20]. Given the safety, cleanliness, and services provided by the initiative, many components of *Project Roomkey* overlapped with trauma-informed care and Health-in-All-Policies approaches, even if not explicitly stated [21]. Sacramento County coordinated placements in its region and first prioritized rooms for those at higher risk of severe COVID-19 infection, including age and comorbidity considerations. These decisions were guided by emerging evidence at the time. To learn about priorities and engagement with services in Sacramento, volunteer medical students gathered feedback from those served by administering surveys and conducting in-depth interviews with unsheltered individuals living on the street or in encampments (“outside PEH”) and individuals who were able to access the emergency hotel room placements (“hotel PEH”). We hypothesized that accessing a hotel placement would relate to increased access to primary care. To inform a Health-in-All-Policies approach to future disasters, the objective of this study was to assess the impact of the disaster response on the unhoused communities surrounding Sacramento. In integrating an interpretation of both individual perceptions and significant group differences between outside and hotel PEH, this study aspires to add to the literature by producing recommendations in how to optimize outcomes for PEH in a disaster.

## Methods

### Participants

Study participants included adult PEH living outdoors on the street or in encampments (“outside PEH”) and adult PEH living in temporary hotel housing as part of Project Roomkey (“hotel PEH”).

### Study Design

**Fig. 1:**
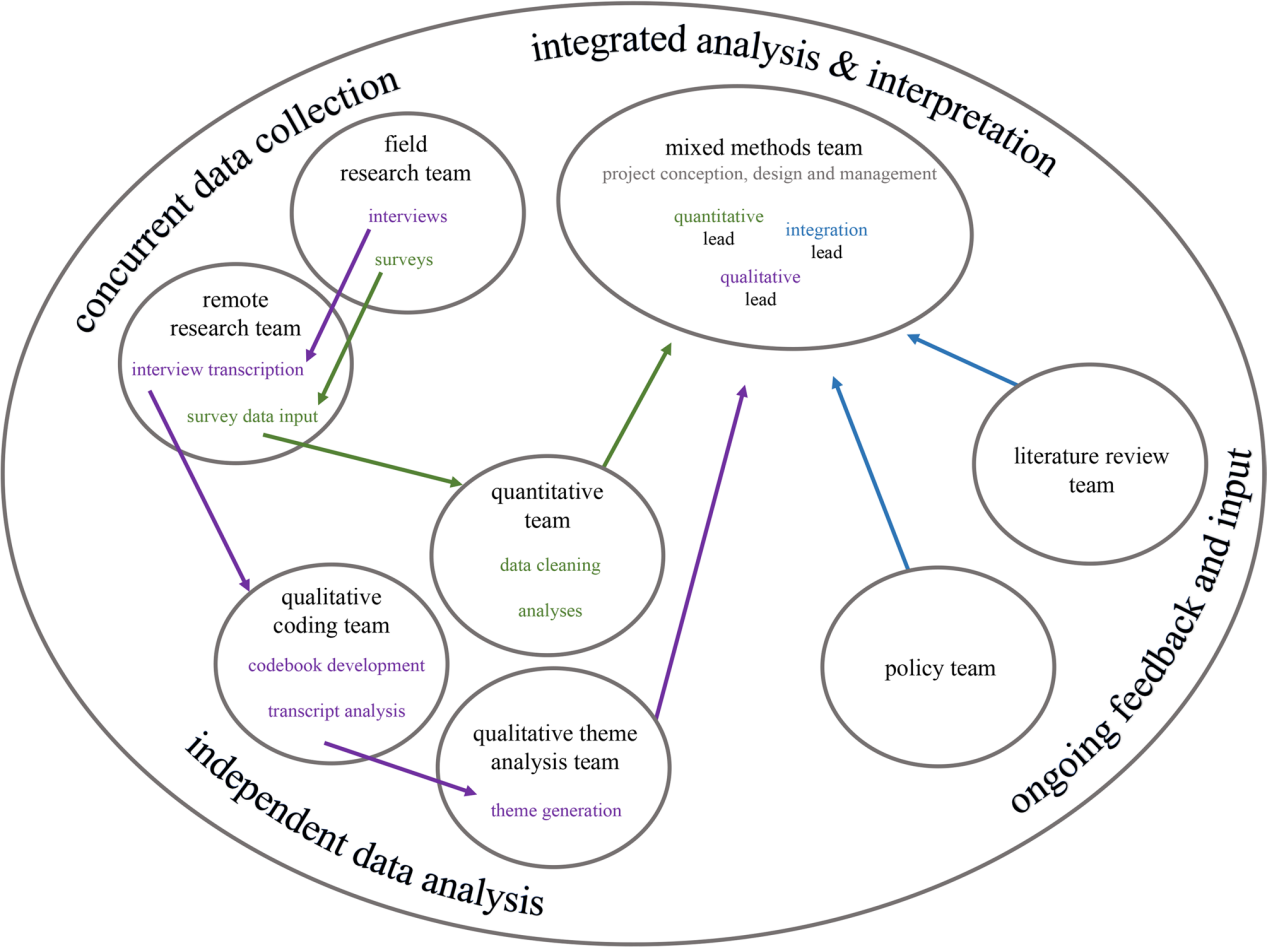
Team-based mixed methods study design. The project was co-led by the mixed methods team, involving the integration lead, quantitative lead, and qualitative lead. Qualitative (purple) and quantitative (green) data collection occurred concurrently with initial independent analyses. Integration (blue) involved incorporation of findings from the initial quantitative and qualitative analyses, additional quantitative reporting utilizing metrics generated from the qualitative analysis process, and perspectives from the policy and literature review teams. See Additional file 1 for an overview of student researcher involvement per team

The study protocol was approved by the University of California, Davis Institutional Review Board (IRB). This study follows the Consolidated Criteria for Reporting Qualitative Research (COREQ) reporting guidelines [22], the American Association for Public Opinion Research (AAPOR) reporting definitions for survey studies [23, 24], and the Strengthening the Reporting of Observational Studies in Epidemiology (STROBE) recommendations [25]. This convergent parallel mixed methods study included quantitative and qualitative data collection with independent analyses, followed by an integrated interpretation and synthesis (Fig. 1) [26]. We employed a team-based approach to multiple phases of the research process and involved a total of twenty-four medical students representing two Northern California medical schools and one undergraduate student [27]. Based on student researchers’ availability, skillsets, and interests, they participated on one or more of the following teams: field research (facilitating surveys and/or interviews), remote research (involving survey data input and/or interview transcription), quantitative (participating in data cleaning and/or analysis), qualitative coding (including codebook development and/or transcript analysis), qualitative theme analysis (generating themes), policy (identifying connections to policy changes), literature review (incorporating knowledge from prior studies), and mixed methods (involving integrated analysis and interpretation) (Fig. 1). See Additional file 1 for number of researchers involved per team.

### Data Collection Procedure

During the emergency response to the COVID-19 pandemic, volunteer medical students, the “Encampment Med Team,” were sworn in as Disaster Service Workers in Sacramento County on March 27, 2020, and worked on the streets and in encampments to raise awareness about COVID-19, increase access to services including testing and supplies to prevent disease transmission, and provide a bridge to hotel housing. Data collection was conducted by a trained student researcher team, many of whom were also part of the “Encampment Med Team,” and researcher visit days occurred in May and June 2020 throughout the Sacramento region at six outside street and encampment sites and at four hotels participating in pandemic relief housing programs. The outside locations were chosen because, (1) they had a weekly Disaster Service Worker medical student volunteer serving as an outreach site lead, and (2) there was a high proportion of unsheltered individuals staying in or passing through that area in comparison to other parts of Sacramento.

Prospective outside PEH participants were informed of the researcher visit day by the student outreach site lead. All original hotel locations dedicated to *Project Roomkey* in the Sacramento area were included in the study, and hotel staff informed prospective hotel PEH of the researcher visit day. Prospective participants voluntarily agreed to learn more about the study during outside site and hotel site researcher visit days. Prospective participants were informed that: (1) the research team was interested in understanding how the disaster response was affecting the unhoused population and how this experience could inform future disaster response policies, and (2) that it was both optional and voluntary to participate in the study, and that the decision to participate would not affect outreach or housing services.

Members of the research team administered standardized surveys followed by optional semi-structured interviews to the same research participant, for subsequent quantitative and qualitative analyses, respectively. For those who agreed to participate in both components of the study, survey and interview were completed in the same encounter. To be considered for the quantitative and/or qualitative study, participants were required to provide informed consent, communicate in English, and be 18 years of age or older. Participants were given a $5 gift card after successful completion of the survey and an additional $5 gift card after successful completion of an interview. Participant responses were assigned a unique identifier number to maintain confidentiality.

### Survey Methods and Data Analysis

For the quantitative assessment, a cross-sectional survey was administered to 100 outside PEH and 100 hotel PEH. The main exposure variable was the dichotomous variable housing placement: outside or hotel. The primary outcome was the number of self-reported visits to the primary care physician. The secondary outcomes included self-reported dichotomous variables (e.g., “Yes/No”) including reported medication adherence, access to a COVID-19 test, access to sanitation and housing related services, use of student services in the field, and access to food. All participants were provided an opportunity to specify their sex, gender and preferred pronouns. The final survey can be seen in Additional file 2. We calculated a modified comorbidity index using the Deyo, Romano, and Glasheen adaptations of the Charlson comorbidity index [28–30] (for the exact scoring approach used, see Additional file 3).

For sample size calculations, 30 days was assumed to be one month for math simplification. Based on previous literature, the number of primary care physician visits per month ranged from 0.3 to 0.6 in unsheltered individuals receiving usual care as opposed to a more comprehensive intervention [31, 32]. Using the conservative estimate of 0.3 baseline primary care physician visits per month, an α of 5% and power of 80%, a sample size of 88 in each arm was calculated to detect a 100% increase (incidence rate ratio [IRR] of 2) of primary care physician visits per month. This assumed a moderate association between the predictor and the other covariates (R-squared: 0.2). Adjusting for a possible 10% in missing data, we estimated a sufficient sample size to be 100 in each arm.

Surveys were guided by a paper instrument with trained survey administrators to increase comfort in interviewing this vulnerable population. Surveys were administered verbally. Data were inputted into a Research Electronic Data Capture (REDCap) database and checked for accuracy by 2–3 other research team members. In bivariate analyses, *t*-tests and chi-square tests were utilized as appropriate. Missing values were imputed using the sample mode for categorical data and sample median for continuous data. Covariates were identified from literature for the multivariable model. Negative binomial models were used to calculate IRRs and confidence intervals (CIs) because the distribution of primary care physician visits was overdispersed during initial data exploration. For medication adherence, odds ratios were calculated from a logistic regression among the individuals who self-reported taking a prescribed medication. We controlled for comorbidity index [33–35], age [36, 37], and sex [36, 38] a priori because they are known predictors of primary care physician care utilization. For this study, a *p-*value less than 0.05 was considered significant. Data preparation and statistical analyses were conducted using R Version 3.5.2 and Stata 16 [39, 40].

### Interview Methods and Data Analysis

Qualitative data collection involved a semi-structured interview designed to elucidate a subject’s pandemic experience using open-ended questions regarding COVID-19 knowledge and attitudes, experience with access to health care, attitudes toward public health interventions, and personal priorities (Additional file 4). Candidates were recruited after having completed the quantitative survey based on their attention level and willingness to communicate verbally. All outside and hotel interviews were conducted by a student researcher in an outdoor space (ex. street, encampment, hotel courtyard), as private as possible, and the interviews were audio-recorded. Recordings were later transcribed by a student researcher on the remote team. Twenty-five outside PEH and forty-seven hotel PEH participated in the interviews. Since all participants were interviewed in outdoor spaces, many transcriptions were limited in length and/or quality due to significant portions of inaudible dialogue when there was excess background or ambient noise, like too much wind. Transcripts were grouped as short, mid-length and long; and only mid-length and long transcripts with minimal ambient noise were selected for the coding team. Of all audio-recorded interviews, nineteen (outside) and sixteen (hotel) of these most substantive and representative interviews were included for full transcription, coding, and thematic analysis.

The first and second authors, two medical students who participated in a volunteer health care team for outside PEH, led data collection and interview administration, and generated an initial codebook based on Strauss and Corbin’s grounded theory approach [41]. Then, they trained a qualitative team of 14 students who committed to learning the coding system. Students were paired up and assigned transcripts to initially code individually and then discuss in pairs to reconcile any differences before meeting with the main team. Transcripts were coded using MAXQDA 2020. Using a recursive, iterative, open coding process, the qualitative team revised the codebook [23, 24]. Each updated codebook was then used for subsequent assignments, and the finalized codebook was utilized to re-code all transcripts.

Subthemes emerged throughout the entire process and were captured in memos. Additionally, after coding was completed, 8 members of the original qualitative team generated further questions to increase understanding of the subthemes. The question-subtheme concepts were further analyzed by individual members and then reviewed by the larger team, where main subthemes were chosen based on recurrence, relation to the primary hypothesis, and significance for policy decisions. We created 4 themes to sort and relate the subthemes. Finally, a draft explaining the themes was compiled and presented back to the qualitative team for final review for unsubstantiated interpretations and bias control. To further characterize code use and influence on theme and subtheme formation, we retrospectively looked at the number of transcript analyses that included the use of each code.

### Integration Methods and Data Analysis

Analytical and interpretive integration was applied by adapting the “following a thread” method, which provides a framework to take the initial analysis of multiple datasets and investigate findings which relate to the overarching research objective [42]. This integrative approach simultaneously preserves an exploratory qualitative process and the focused specificity of a quantitative process [43]. Starting with a grounded inductive approach, an analytic question/theme (thread) was first defined based on the current study and insights from the literature team and policy team. The thread was followed across datasets, iteratively assessing the data through new lenses, to generate a multifaceted picture addressing the analytic question. Results from the first thread led to the identification of four emerging threads, which were followed up in a similar fashion of iterative data interrogation. Data transformation was incorporated to generate guideline-based recommendations [44]. Then, to probe an ultimate thread representing the entire study objective, results from all five threads were treated as individual datasets and used in a meta-analysis, resulting in an integrated interpretation. Visual joint displays as figures rather than tables were utilized to illustrate the complexity of data integration as relating to the thread, specifically quantitative and qualitative data results for the first thread, and all five initial threads for the meta-analysis thread.

## Results

### Quantitative Results

Among the six encampment and street sites and four hotels/motels, 200 surveys were collected: 100 from outside PEH and 100 from hotel PEH. Outside PEH were younger, with a median age of 48, as compared with a median age of 56 for hotel PEH (Table 1, placed before references due to length). In comparison to outside PEH, hotel PEH had significantly more self-reported non-psychiatric chronic medical conditions (87% vs. 66%) and psychiatric conditions (61% vs. 37%). Outside PEH were more likely to report polysubstance use (81% vs. 58%) and previous incarceration (36% vs. 20%) than hotel PEH. Pet ownership was common for both populations, with 44% of outside PEH and 29% of hotel PEH owning at least one pet. There were no significant differences with respect to self-reported primary language, sex, or race and ethnicity. Not all individuals who identified their sex as male or female identified their gender as male or female, respectively, and pronoun preferences were noted (see Additional file 5).

**Table 1:** Baseline Characteristics by Housing Location n (%) (unadjusted)

Variable	Outside PEH *n* = 100	Hotel PEH *n* = 100	*p*-value
**Participant Characteristics**			
Patient age in years, median (IQR)	48 (18–68)	56 (19–78)	< 0.001
Sex			0.08
Male	59 (59%)	48 (48%)	
Female	36 (36%)	51 (51%)	
Something Else/Unknown	5 (5%)	1 (1%)	
Race/Ethnicity			0.76
Hispanic	8 (8%)	8 (8%)	
Non-Hispanic White	36 (36%)	37 (37%)	
Non-Hispanic Black	25 (25%)	25 (25%)	
Non-Hispanic Native American	7 (7%)	4 (4%)	
Non-Hispanic Asian/Pacific Islander, Other, Missing	6 (6%)	11 (11%)	
Mixed	18 (18%)	15 (15%)	
Veteran	25 (25%)	8 (8%)	0.47
Primary Language			0.45
English	95 (95%)	98 (98%)	
Other or Unknown	5 (5%)	2 (2%)	
**Medical Characteristics**			
Insurance			0.19
Public	62 (62%)	71 (71%)	
Private	14 (14%)	15 (15%)	
Unknown	24 (24%)	14 (14%)	
Comorbidity Index, median (IQR)	0 (0–0)	1 (0-1.3)	< 0.001
Psychiatric Condition	37 (37%)	61 (61%)	0.001
Non-Psychiatric Chronic Medical Condition	66 (66%)	87 (87%)	< 0.001
BMI, median (IQR)	25 (23–29)	27 (23–33)	0.07
			0.03
Underweight	2 (2%)	4 (4%)	
Normal Weight	41 (41%)	30 (30%)	
Overweight	40 (40%)	32 (32%)	
Obese	17 (17%)	34 (34%)	
**Social History**			
Tobacco Smoker			0.36
Current	62 (62%)	71 (71%)	
Former	14 (14%)	15 (15%)	
Never or Unknown	24 (24%)	14 (14%)	
Marijuana Use	64 (64%)	43 (43%)	0.005
Intravenous (IV) Drug Use	14 (14%)	4 (4%)	0.02
Methamphetamine Use	43 (43%)	10 (10%)	< 0.001
Alcohol Use	57 (57%)	33 (33%)	0.001
Reported Polysubstance Use (2+)	81 (81%)	58 (58%)	< 0.001
Previous Incarceration	36 (36%)	20 (20%)	0.02
Ownership of One or More Pets	44 (44%)	29 (29%)	0.04
Number of Pets			0.01
0	56 (56%)	71 (71%)	
1	25 (25%)	18 (18%)	
2	12 (12%)	11 (11%)	
3+	7 (7%)	0 (0%)	
Household Size			0.46
1	66 (66%)	64 (64%)	
2	22 (22%)	28 (28%)	
3+	12 (12%)	8 (8%)	
Duration of current location of residence^1^, median (IQR)	7.0 (2.1–24)	1.1 (0.6-2.0)	< 0.001
Number of Relocations in Past Month^2^	1 (0–3)	1 (0–4)	0.46

*PEH* Person/People Experiencing Homelessness

^1^ Missing: 0 in Outside PEH, 4 in Hotel PEH

^2^ Missing: 1 in Outside PEH, 4 in Hotel PEH

#### Primary Outcome: Primary Care Physician Access

More hotel PEH participants reported having a primary care physician than in outside PEH (60% vs. 48%), but this difference was not statistically different (*p* = 0.13). Hotel PEH had a higher median number of primary care physician visits in the last month than outside PEH in unadjusted analyses (Table 2). As seen in Table 3, after adjusting for age, sex, and comorbidity index, hotel PEH was associated with a greater number of reported visits in the past month than outside PEH (IRR: 2.16, 95% CI: 1.08–4.36). Outside PEH had an estimated average of 0.34 (95% CI: 0.14–0.54) visits in the past month and hotel PEH had an estimated average of 0.73 (95% CI: 0.41–1.05) visits in the past month.

**Table 2.** Reported Utilization of Health Care and Other Services, *n* (%) (unadjusted)

	**Outside PEH** *n* = 100	**Hotel PEH** *n* = 100	***p*** **-value**
**Use of Health Care Services**			
Primary Care Physician	48 (48%)	60 (60%)	0.13
Primary Care Physician Visits in Last Month, median (IQR)	0 (0–1)	1 (0–2)	0.051
Received a COVID-19 Test			< 0.001
True	27 (27%)	60 (60%)	
False	64 (64%)	36 (36%)	
Missing	9 (9%)	4 (4%)	
Access to…			
… working hand-washing station	72 (72%)	100 (100%)	< 0.001
… bathroom	69 (69%)	100 (100%)	< 0.001
… hand sanitizer	87 (87%)	73 (73%)	0.01
… face mask	65 (65%)	91 (91%)	< 0.001
… shower	54 (54%)	100 (100%)	< 0.001
… electricity	35 (35%)	98 (98%)	< 0.001
**Use of Student Outreach Services** ^1^			
Mask	60 (60%)	46 (46%)	0.07
Hand-washing station	71 (71%)	42 (42%)	< 0.001
Hand sanitizer/Soap	71 (71%)	49 (49%)	0.002
COVID-19 Test	35 (35%)	38 (38%)	0.77
COVID-19 Information	60 (60%)	33 (33%)	< 0.001
Food	81 (81%)	52 (52%)	< 0.001
Street clinic	34 (34%)	20 (20%)	< 0.001
Telemedicine	14 (14%)	7 (7%)	0.17
Wound care	45 (45%)	22 (22%)	0.001
Link to housing	27 (27%)	37 (37%)	0.001
Other^2^	10 (10%)	4 (4%)	0.17
None	2 (2%)	33 (33%)	0.001
Total Student Services Accessed (Median, IQR)	5 (4–7)	3 (0–6)	0.001
**Other**			
Number of daily meals, median (IQR)	2 (1.5-3.0)	3 (3–3)	< 0.001
Balanced Meal^3^ for Previous Dinner	20 (20%)	60 (60%)	< 0.001
Number of People Sleeping within 10 feet of interviewee^4^, median (IQR)	1 (0–3)	0 (0–1)	< 0.001
Close Contacts in Past 24 Hours^4^, median (IQR)	6 (3–15)	2 (1–6)	< 0.001
If you could, would you…			
… get into a hotel room/stay in a hotel room?	91 (91%)	96 (96%)	0.17
… use services of a mobile clinic?	93 (93%)	85 (85%)	0.07

PEH = Person/People Experiencing Homelessness

^1^ An interviewee’s self-reported use of outreach services provided by medical student Disaster Service Worker volunteers visiting street and encampment sites. For hotel PEH, this was specified as prior to hotel placement.

^2^ Other included wipes, psychological services/mental health (*n* = 2), water (*n* = 5), animal food, needle exchange, veterinarian, haircut, hospital trip

^3^ Balanced meal considered to be a meal with protein, vegetables and grain/carbs.

^4^ Missing: 4 in Outside PEH, 1 in Hotel PEH

#### Secondary Outcomes

Hotel PEH were more likely to have received a COVID-19 test (Table 2), and all individuals who received a COVID-19 test were self-reported negative. Access to shelter and hygiene-related services were generally higher for hotel PEH than outside PEH. All participants (100%) at the hotel reported having working hand-washing stations, bathrooms, showers, and electricity compared to the 72%, 69%, 54%, and 35% of outside PEH. Similarly, hotel PEH reported a higher median number of daily meals and were more likely to report having a balanced meal for the previous dinner before the interview. Surveyed outside PEH also reported more people sleeping within 10 feet and a greater number of close contacts in the past 24 h.

Also seen in Table 3, hand sanitizer was the only item that outside PEH reported as having increased access (87% vs. 73%, *p* = 0.01). Surveyed outside PEH were more likely to have encountered volunteer pandemic response student services, with the exception of a link to housing which was higher in hotel PEH. Most study participants favorably saw the hotel process (91% outside PEH and 96% hotel PEH) and favorably viewed the idea of accessing health care services by a mobile clinic (93% outside PEH and 85% hotel PEH).

One hundred and thirty-one individuals reported currently taking medications: 47 (36%) outside PEH and 84 (64%) hotel PEH. A greater proportion of individuals reported medication adherence in hotel PEH than outside PEH (70% vs. 45%, *p* = 0.004). After adjusting for age, sex, and comorbidity index, housing at a hotel was associated with greater reported medication adherence (adjusted odds ratio [aOR]: 2.60, 95% CI: 1.21 − 5.56) (Table 3). Outside PEH demonstrated a 52% (95% CI: 38%−66%) probability of medication adherence in comparison to 74% (95% CI: 64% − 84%) of surveyed hotel PEH.

Further, we found that the hotel selection process successfully prioritized individuals with chronic pulmonary disease (5% outside PEH vs. 29% hotel PEH, *p* < 0.001) (Table 4). Although not statistically significant individually, many other conditions tended to be represented at a higher percentage in the hotel PEH population than in the outside PEH population, including diabetes mellitus, kidney disease, liver disease, and cancer. Taken together, and as evidenced by the significantly higher comorbidity index in hotel PEH (*p* < 0.001), the hotel placement process had successfully prioritized those PEH with more complex medical needs.

**Table 3:** Multivariable Models of Reported Primary Care Physician Visits in the Past Year and Reported Medication Adherence

Outcome	Primary Care Physician Visits *n* = 200	Medication Adherence *n =* 131
Variables	Incidence Rate Ratio	Odds Ratio
Location		
Outside PEH	Ref.	Ref.
Hotel PEH	2.16 (1.08–4.36)*	2.60 (1.21–5.56)*
Age	1.01 (0.98–1.04)	1.01 (0.98–1.05)
Sex		
Male	Ref.	Ref.
Female	0.74 (0.36–1.51)	1.21 (0.56–2.64)
Comorbidity Index^1^	1.09 (0.82–1.45)	1.48 (1.05–2.09)*

PEH = Person/People Experiencing Homelessness; ^*^ *p* < 0.05, ^**^ *p* < 0.01, ^***^ *p* < 0.001

^1^ Modified version of Charlson’s Comorbidity Index, see Additional file 3

**Table 4:** Reported Conditions and Comorbidity Index

	**Outside PEH** *n* = 100	**Hotel PEH** *n* = 100	***p*** **-value**
**Comorbidity Index**			< 0.001
0	72 (72%)	39 (39%)	
1	14 (14%)	34 (34%)	
2	7 (7%)	15 (15%)	
3	5 (5%)	7 (7%)	
4	1 (1%)	4 (4%)	
5	1 (1%)	0 (1%)	
6	0 (1%)	1(1%)	
**Reported History of…**			
Cerebrovascular Disease	0 (0%)	2 (2%)	0.49
Chronic Pulmonary Disease	5 (5%)	29 (29%)	< 0.001
Rheumatologic Disease	3 (3%)	3 (3%)	1.00
Diabetes Mellitus	13 (13%)	20 (20%)	0.25
Kidney Disease	5 (5%)	11 (11%)	0.19
Liver Disease	6 (6%)	11 (11%)	0.31
Cancer	3 (3%)	10 (10%)	0.09
HIV	3 (3%)	0 (0%)	0.25
**Reported Psychiatric Conditions**			
Depression	17 (17%)	25 (25%)	0.22
Anxiety	7 (7%)	17 (17%)	0.050
Schizophrenia, Schizoaffective Disorder	9 (9%)	10 (10%)	1.00
Bipolar Disorder	13 (13%)	22 (22%)	0.14
Post-Traumatic Stress Disorder	6 (6%)	10 (10%)	0.43
Other^1^	7 (7%)	13 (13%)	0.24

^1^ Other included dissociative disorder, attention deficit hyperactivity disorder, obsessive-compulsive disorder, personality disorders such as borderline

#### Qualitative Results

We sought to assess how unsheltered individuals experienced the COVID-19 pandemic emergency response as related to socioeconomic and environmental factors contributing to health. Interviewees were asked to describe their perspectives of these factors – including access to information on COVID-19, primary care, public services, interaction with law enforcement, and interaction with other unsheltered individuals – and how they felt in terms of housing status, identity, fears in life, and most important need. See Additional file 4 for a comprehensive list of interview questions.

Twenty-five outside PEH and forty-seven hotel PEH participated in the interviews. Of the transcribed audio-recorded interviews, thirty-five of the most substantive and representative were included for full transcription, coding, and thematic analysis. These transcripts included nineteen from outside PEH and sixteen from hotel PEH.

We initially generated 62 codes, and through 3 subsequent rounds of coding and codebook discussions, the codebook was expanded and refined to 77 codes, of which 11 are modifiers (e.g. “weak”). Modifiers provided a way to emphasize a code. The final codebook with code descriptions appears in Additional file 6.

Several subthemes were identified: information, sanitation, community actions, trust with providers, chronic health conditions, sweeps, barriers to housing, and housing access. Examples of interview segments categorized by subtheme appear in Additional file 7. These subthemes were organized into 4 themes: (1) Access to resources, relating to availability affected by socioeconomic and ecological context; (2) Social connection as empowerment, relating to relationships with institutions and others in the housed community; (3) Exacerbation of pre-existing conditions, relating to any physical or mental conditions that existed before the pandemic; and (4) Impact of systems and policy on safety, relating to the decision or action of a government body (Table 5). Representative quotes that appear in the main text as related to these core four themes are summarized in Additional file 8.

Separately from the coding process and thematic analysis, we retrospectively sought to quantitatively describe code usage by researchers by noting the number of transcripts analyses involving the use of each code (Additional file 9). Codes used in a high proportion of all transcripts showed clear overlap with the finalized themes and subthemes. For example, the code BASIC NEEDS: NETWORK – relating to comments on networks, relationships, family, relatives, and friends – was used in 35/35 (100%) of transcript analyses, indicating importance in the formation of the second theme, among others.

**Table 5:** Qualitative Analysis

Themes	Subthemes
Access to Resources	Information Sanitation
Social Connection as Empowerment	Sanitation as a Community Action Trust with Providers
Exacerbation of Pre-Existing Conditions	Chronic Health Conditions
Impact of Systems & Policy on Safety	Sweeps Barriers to Housing Housing Access

### Theme: Access to Resources

An important theme that emerged was access to resources that enable integration into society, especially as affected by socioeconomic and ecological contexts. In the absence of a global pandemic, PEH at baseline had limited resources. As one 62-year-old man (he/him) living outside put it:

I’m homeless. I got nothing. What you see here is something that I’ve gathered up due to a lot of people giving stuff up and go from there. Some of it’s in good shape, some of it’s not. You do what you can.

Closures during the pandemic emergency response exacerbated preexisting scarcities. Key subthemes explored included access to reliable information on current events and access to basic needs like a sanitary living environment. The codes ACCESS, INFO, and BASIC NEEDS: SANITATION were utilized in 91%, 97%, and 94% of all transcripts, respectively (Additional file 9), indicating commonly discussed topics.

#### Information

Lack of information about the pandemic was prevalent in outside PEH, while less so at the hotels, as one 52-year-old man (he/him) interviewed at a hotel explained:

It’s hard to get any information. I’ve got more information since we’ve been here [at the hotel] and I’ve watched the news. The homeless can’t just watch the news. Some have a radio, but most don’t.

Without access to television, the internet, or regular healthcare providers in the encampments as compared to the hotels, misinformation ranging from anti-government conspiracy to beliefs of immunity in PEH abounded in the encampments. Hotel PEH did not always have accurate information either, as a 51-year-old man (he/him) in a hotel described:

I got a text from uh, a lady friend, she did a chain text, and she said, ‘Hold your breath 10 seconds in the morning, and if you don’t start coughing real bad after 10 seconds of holding your breath in this area, then you don’t have it.’ And I been doin’ that ever since I read that.

Access to information was needed, not only for COVID-19 prevention and testing, but also for navigating social services when most physical offices had closed. A 57-year-old man (he/him) at a hotel described the mazelike system of obtaining social benefits and how COVID-19-related closures had increased barriers:

Now [if] you don’t have a driver’s license, you need an ID…you need [your] birth certificate…then it just becomes hard…you have no access to them…the office is not open.

These systems, already challenging without shelter-in-place, only became more difficult to engage with for PEH who lacked access to reliable transportation, internet, or phones.

#### Sanitation

Both outside PEH and hotel PEH connected their sanitation access to their ability to protect themselves from COVID-19. While some outside PEH worked assiduously to maintain some hygiene, others professed a substantial degree of fatalism when asked how they planned to prevent disease spread. One 56-year-old woman (she/her) who had accessed hotel housing explained how it was like living outside:

And now it’s finally caught up with us. And we were dirty, we were not being clean enough. C’mon that’s what this pandemic is all about. It’s clean-li-ness. Homeless people can’t be washing their hands every 20 min. It’s impossible.

In contrast, hotel participants were more optimistic about their hygiene and commented on particular actions taken to improve their personal hygiene and protect themselves from potential transmission. Many expressed gratitude for individual showers and sinks.

Though pop-up sanitation stations were added due to COVID-19, interviewees living outside described decreased overall access to services due to closing businesses and public areas. Multiple respondents reported that the shutdown of restaurants and public parks had severely impacted their access to a bathroom. One 60-year-old woman (she/her) living outside said,

You can’t get access. You can’t use the public bathrooms anymore.

A 50-year-old woman (she/her) shared her experience living outside before hotel placement, noting:

You have all these people downtown, homeless with no restroom. And we kept hearing that they were gonna put washing stations in the park across the street – which they did – but they didn’t maintain them. We were just kind of lost down there.

Without regular access to running water, bathrooms, and showers, efforts to prevent disease transmission are impaired. Their responses highlighted the patchwork nature of resources PEH rely on for basic sanitation needs and the complexity of maintaining access amidst a pandemic.

### Theme: Social Connection as Empowerment

A second theme that emerged was the importance of social connection as a source of empowerment, including relationships with housed community members, other PEH, health providers, and institutions.

Empowerment relates to the ability of an individual to have a sense of agency as an active participant in one’s actions, like being in the driver’s seat of one’s own life situations [45]. The code POWER: EXTERNAL, relating to the belief that an external power has agency, was utilized in 91% of all transcripts (Additional file 9), indicating it was commonly discussed. Sometimes, the interviewee felt disempowered since a larger entity had control of a life situation. Nonetheless, the code POWER: SELF, relating to the belief that the interviewee has agency, was also commonly discussed, being utilized in 83% of all transcripts (Additional file 9). Thus, depending on the context, interviewees demonstrated an understanding that they had agency in a life situation. For example, the ability to recognize one’s contributions to a healthy social network and community can enable individuals to care for themselves as interconnected with caring for the community. One 57-year-old man (he/him) interviewed in a hotel reflected on the importance of self-care and self-love for preventing disease spread, explaining:

Whatever can happen to you can happen to me. If I’m sick [with the virus] and I don’t take care of myself, you can get sick [through transmission of the virus]. So, it’s like people have to love themselves.

People who experience homelessness are a heterogeneous population, as everyone has a unique life story. A 43-year-old woman (she/her) interviewed outside reflected on how making assumptions about a person based on life circumstances can limit exposure to incredible people:

A lot of people look down on being homeless. A lot of people look at us like the scum of the earth sometimes. I have met some of the greatest people – who are homeless. Some of the people with houses, not so beautiful. I do say money is the root to all evil. So, being broke has never been my problem… When they judge a book by its cover they might miss a great story.

Respect and compassion underpin empowering interactions. Key subthemes explored included sanitation as a community action and trust with providers, both of which can relate to empowering social connections. The codes BASIC NEEDS: NETWORK, IDENTITY, and TRUST were utilized in 100%, 100%, and 86% of all transcripts, respectively (Additional file 9), indicating commonly discussed topics.

#### Sanitation as a Community Action

For both outside and hotel PEH, the discussion often revealed that respondents’ engagements with sanitation were influenced by cleanliness being entwined with a sense of dignity and a sense of community and inclusion. For example, a 42-year-old woman (she/her) living outside poignantly described how she felt about judgment around lack of personal hygiene:

We are considered the lost, the not worthy of anything in life…we are treated as garbage. I have physical conditions that make it hard for me to even be able to use the restroom with some form of dignity and then feel like there is some kind of sanitation.

If dignity seems lost with uncleanliness, an empowering community action would be helping clean, such as removing garbage from shared spaces. Many unhoused individuals discussed ongoing intra-community efforts to maintain their hygiene and sanitation, and they also described positive experiences where service providers entrusted resources and responsibility towards these efforts. A 49-year-old man (he/him) living outside shared:

For a while, I got known to where, you know, I’ve had park rangers give me trash bags ‘cause they’d see me picking up trash, and now if every homeless person did that, we wouldn’t get fines…They’d see that eh – at least we’re, you know, doing a good job keeping everything clean.

Hotel respondents expressed pride at cleaning communal spaces and directly related these actions to positive interactions and relationships with staff and service workers. Several PEH further expressed hopes that future plans and programs would take deliberate steps to empower their community hygiene needs with resources and support.

#### Trust with Providers

When asked about successful services such as testing, health care, and even hotel housing, outside PEH respondents often made the point of explicitly naming or calling attention to the provider to whom they attributed the positive interaction and with whom they had developed rapport. These trusted providers were often members of grassroots health initiatives that emerged to address the unique needs of testing and triaging unhoused populations during the pandemic. Healthcare professionals and students who were familiar with encampment communities through previous volunteer and professional work were recognized and seen differently. A 65-year-old man (he/him) interviewed in a hotel explained:

What was most important – what hit my heart? The [students] would come around to the homeless people, on their own time, and offer hand sanitizer and ask you what’s wrong with you, if you need any help: Do you need any suggestions on where to go or what to do? That has probably meant the most.

Distrust of the medical community was a common theme among outside PEH respondents, and many expressed doubts over testing and other health services from unknown providers. Having familiar outreach workers and providers deliver health education and encourage uptake of services was significant in respondents’ depiction of their decisions to engage. One 33-year-old woman (she/her) living outside explained:

I don’t really talk to them [unfamiliar outreach workers]. I talk to you guys.

People in the hotel spaces were less likely to name specific health care providers, often referring to the providers that visited the site as “the doctors.” Among those interviewed at the hotels, while general distrust of systems was mentioned, none described mistrust of their provided health care. For both outside PEH and hotel PEH, routine social connection with familiar and reliable providers enabled ongoing care, empowering individuals to care for themselves and those around them.

### Theme: Exacerbation of Pre-existing Conditions

A third theme that emerged was the worsening of pre-existing conditions, relating to any physical or mental condition that existed before the pandemic. The key subtheme explored was in relation to chronic health conditions. Management of chronic conditions could be tricky for PEH, even without a pandemic, as one diabetic 57-year-old man (he/him) reflected on the challenges of managing his sugar levels with insulin without predictable meals:

It’s not just the epidemic, it’s my health. So I’m not trying to blame corona[virus], I blame my health and yeah you blame it on yourself for not taking care of it. … You have to take your medication before your food. But if you dunno what food you’re gonna eat, how are you gonna take your medicine? So I wouldn’t take my medicine then….

The codes HEALTH: HEALTHCARE (discussion of interaction/perspective with healthcare), HEALTH: PREVENT (discussion of preventative care), and HEALTH: SELF-RISK (discussion of self-perceived probability of harm) were utilized in 100%, 91%, and 83% of all transcripts, respectively (Additional file 9), indicating commonly discussed topics.

#### Chronic Health Conditions

While those in the hotel had reliable access to electricity, many felt that their needs for medication management and health care for chronic conditions were still unmet. A 53-year-old woman (she/her) staying at a hotel shared how specific services could help improve her situation:

I could use a social worker, and I really need help with my medications. If I did get the virus, that would be scary. … Transportation is a huge issue for me. I wasn’t able to make it to my doctor’s appointment.

Both outside PEH and hotel PEH criticized the lack of consideration felt to have been taken for the population’s chronic health conditions and unique reliance on communal spaces. For example, the shutdown of communal kitchens provided by local nonprofits took away many interviewees’ only reliable option for medication refrigeration and storage. A 36-year-old diabetic interviewee (no specified pronouns) living outside explained:

I actually had to turn down my insulin because of the fact that I have no reason or no way to keep it cold no more ‘cause I [now] have no access to the ice machine.

The same interviewee, who managed multiple comorbidities, further commented on how limited access to reliable medical and lab services affected medication management and disease progression:

I’m epileptic; and I can only get one of my three seizure meds because the other two have to be monitored, and so I go through a lot of seizures a day.

Treatments for substance use disorders were also severely impacted, including the shutdown of in-person Alcoholics Anonymous meetings and therapy. While several of these services had shifted online, many respondents explained that they could not consistently access technology or the electricity needed to run their electronics. Several disclosed that they had friends who relapsed during this gap in access. Harm reduction services were also less frequently available during the pandemic since many outreach providers were constrained from regular visits. With the absence of community resources, some respondents reported taking the community’s health into their own hands, such as individuals carrying and administering Narcan to fellow community members.

### Theme: Impact of Systems & Policy on Safety

An integral theme that emerged was the impact of systems and policy on safety, relating to the decision or action of a government body. The code FEAR was utilized in 97% of transcript analyses (Additional file 9), indicating a commonly discussed topic. Some of this was related to safety about housing instability, which was affected by ongoing sweeps by law enforcement and barriers to housing access. The codes HOUSING: HOUSE, PRIORITY, and PUBLIC SERVICES: POLICE were utilized in 89%, 91%, and 89% of transcript analyses, respectively. In California, government resources dedicated to emergency hotel room placements facilitated access to safety and shelter for many PEH. Nonetheless, specific rules and restrictions around this process became barriers for some PEH in accessing shelter and the protection that comes with it. As such, key subthemes explored included sweeps and interactions with police, barriers to housing, and housing access.

#### Sweeps

Policies regarding sweeping of areas with PEH were unclear and unevenly enforced. Some outside PEH reported no sweeps due law enforcement respecting the Centers for Disease Prevention and Control shelter-in-place guidance [46]. In other areas, PEH reported regular encounters with law enforcement, ranging from auditory interruptions to full sweeps and destruction of property.

Of all interviewees, 31/35 (89%) mentioned interactions with police, and these responses were coded as positive, negative, or mixed/neutral (Table 6). Two outside PEH and two hotel PEH described positive experiences, and two of each group did not mention any interactions.

**Table 6:** Perspectives on Experience with Police

	Positive	Mixed/Neutral	Negative	No Mention	Total
Outside PEH	2	7	8	2	19
Hotel PEH	2	8	4	2	16

Seven of the 19 (34%) outside PEH and 8/16 (50%) hotel PEH had mixed or neutral feelings about their interactions with police. Outside PEH had a higher proportion of respondents with negative experiences than hotel PEH, with 8/19 (42%) and 4/16 (25%), respectively.

A 43-year-old woman (she/her) living outside described her experience with sweeps and how continuous forced movement hindered planning for the future:

As long as we move, we are alright. They do that all the time, on a regular basis. When they say homeless outreach, how they are outreaching is to take [our] property – that’s the biggest thing they do… When we are moved around like that it takes our energies and focus away from doing things that can be more preventative from us being out here. You know what I mean? Finding jobs or places we can be. When they are shuffling us around like that, all that does is exhaust us, tear our health down, and kill us off, little by little, really. Or incriminate us by taking us to jail.

Without stability of an outside home base, PEH’s daily priority becomes survival, and long-term planning is not the focus. PEH desired long-term safety strategies that would interrupt these unsustainable cycles of displacement.

#### Barriers to Housing

Despite the benefits of the rapid rehousing offered to PEH during the pandemic, many barriers still prevented specific interviewed individuals from accessing housing. One family met a representative for hotel housing, but not only were no family residences available, the housing came with restrictions preventing residents from leaving for more than 30 min at a time. The 43-year-old woman (she/her) interviewed outside explained why this was infeasible for her family:

Well, my 18-year-old son has [motions to indicate mental disability], so it’s important that we maintain close contact because we are immediate family. But they also said that we would have to stay inside during the day, which means that he [her husband] and I wouldn’t be able to work…and we didn’t find any housing that fit our family unit. And it’s important to me; it’s the priority for me.

A 56-year-old man (he/him) staying in a hotel shared the challenges he faced in eligibility for housing when he didn’t fit specific criteria:

I get told by the navigators that I’m a hard person to place. The other day, last Friday when I was there, they were like well if you were HIV positive, we could get you in on a medical, if you tried to commit suicide, we could get you in on a psychiatric, if you were using drugs, we could get you in to a drug program, but you don’t have any of those, so we don’t know what to do with you.

Several other respondents were unwilling to leave their pets behind due to a lack of safe places to leave them and the fact that they viewed their pets as part of the family. Consequently, they relinquished their offers for hotel stays. Companion animals were frequently discussed, as the code BASIC NEEDS: PET was utilized in 79% of outside PEH transcript analyses and 75% of hotel PEH transcript analyses.

A 49-year-old man (he/him) living outside described how his animals are integral to his safety:

I got two pets and they’ve saved my life many times…‘cause they’re federal registered service dogs. Through myself training them, and plus just the knowledge of the dogs themselves, they’ve- they’ve stopped a car from running me over. They’ve found me when I got lost, they help me get to my camp sites when I get lost.

One 56-year-old woman (she/her) staying in a hotel reflected on the importance of caring for her dog, even choosing to feed her pet instead of herself:

And there [were] times where my dog ate and that was good enough for me.

Companion animals provided many roles for PEH, including safety and security, a sense of family, emotional support, assistance with disabilities, and a way to demonstrate responsibility and compassion. Housing policies with pet restrictions did not always recognize the importance of companion animals to daily functioning and survival.

#### Housing Access

When asked about their priorities, respondents’ answers typically involved housing. Housing, several explained, was the foundation to several basic needs: stability, mental health, and ultimately, a sense of safety. The contrast between responses from hotel PEH and outside PEH was perhaps most stark when asked about their hopes and future plans. People interviewed in encampments were often terse, even disparaging in their answers. A 59-year-old man (he/him) living outside shared:

I say now the way things are, my most important need is getting some better housing. Like I said, I can’t do that, I got no income, da-da-duh, so it’s… logically no way to even think like that.

Other answers were vague without concrete plans, yet still desiring a change. A 54-year-old woman (she/her) living outside explained her most important need:

That’s to get myself and my mind together, and get up outta here. I’m not in a hole and um I wanna make sure that I um, hey, I’m good. Not that I’m crazy but I’m more than that.

Meanwhile, respondents within the hotels more often elaborated on hopes for the future, as a 52-year-old man (he/him) interviewed in a hotel described:

I been tryin’ to correct my credit. I had good credit back in 2001 and 2 and 3. Then uh, like I said, I- I’m gonna look for a place and by then I should have enough money, and, uh, if I can stay here enough time, have enough to put down a deposit and rent. And I’ll be fine from that point on. I just need that extra money [from Social Security] to get in the place, you know what I’m sayin’.

Housed respondents also expanded on more specific hopes such as being reunited with family once having a safe place to spend time with children and grandchildren. Discussion of a housed space as significant in mental and physical safety recurred frequently, as a 50-year-old woman (she/her) staying in a hotel explained:

We’re indoors … We have showers. We have bathrooms. It’s safe; we’re safe.

Not only were hotel PEH more hopeful, but several respondents also expressed gratitude for ongoing access to housing services such as housing navigators. A 56-year-old woman (no specified pronouns) interviewed in a hotel explained:

[I need a] roof over my head – my own roof. I can’t stay with people. That’s what my navigator is trying to do, find me my own. I think God’s giving me a second chance.

Some respondents had time to reflect on how to make these life shifts more sustainable for all PEH. A 56-year-old woman (she/her) staying at a hotel shared how formal classes for integration into society would benefit many who have been living at the margins:

There needs to be classes to make it more successful for a homeless person to go back to society and be regular. I don’t think that they realize – I’ve seen it, I’ve seen it many times – and then they lose their homes.

Emergency hotel room placements facilitated access to many basic needs, and this allowed several PEH not only the ability to shelter-in-place during the pandemic but also have a pause in living day-to-day for survival and make plans for the future.

### Integration Results

#### Integrated Analysis with Thread 1: Comparing and Contrasting Hotel Access

**Fig. 2:**
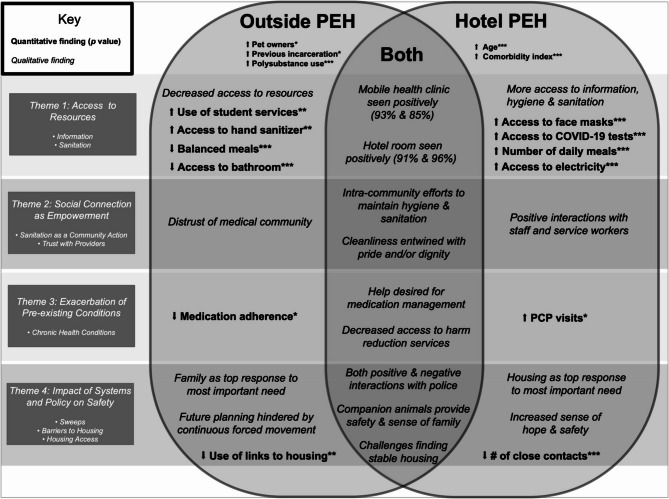
Venn diagram of outside PEH and hotel PEH experiences during the COVID-19 disaster response. Results of Thread 1 integrated analysis comparing results relating to outside PEH and hotel PEH perspectives and experiences, drawing on both quantitative and qualitative assessments. Qualitative findings are in italics to distinguish from quantitative results. Overlap with qualitative themes are color-coded in purple and refer to: (1) Access to resources; (2) Social connection as empowerment; (3) Exacerbation of pre-existing conditions; and (4) Impact of systems & policy on safety. Reported statistics, left-to-right, refer to outside PEH first followed by hotel PEH. For statistically significant differences, findings from quantitative results are indicated; * *p* < 0.05, ** *p* < 0.01, *** *p* < 0.001

Using a grounded inductive approach, we identified the analytic question of Thread 1 - *How do experiences*,* perspectives and priorities relating to the disaster response compare and contrast between outside PEH and hotel PEH?* This inquiry was informed by the literature team, the policy team, the quantitative analytical focus of comparing these groups statistically, and the qualitative finding that housing was a priority. We looked back at interview responses to the question of what the most important need of the interviewee was and categorized and quantified responses (Additional File 10). While housing was a common response for both outside PEH and hotel PEH, outside PEH had more varied responses, with 14 respondents noting needs other than housing, including maintaining connections with family, general hopeful feelings without a concrete plan, and specific items (for ex., blankets). Figure 2 integrates findings from both quantitative results and qualitative results by incorporating qualitative themes, quantitative findings (statistical significance for differences), and qualitative findings. Together, these integrated findings compare and contrast how the emergency response impacted outside PEH and hotel PEH and demonstrate how physical health and socioeconomic and environmental determinants of health are intricately entwined with the impact of systems and policy on safety.

#### Integrated Analyses of Threads 2-5: Generating Guideline-based Recommendations

The pervasiveness of the qualitative themes as relating to both quantitative and qualitative findings led to the identification of four additional investigated threads, all informed by the study objective of incorporating guidelines and recommendations for disaster response. These threads included the following: Thread 2 - *How does a guideline to prioritize housing relate to PEH experiences?*; Thread 3 - *How does a guideline to maintain safe and healthy living environments relate to PEH experiences?*; Thread 4 - *How does a guideline to improve health and access to integrated health care services relate to PEH experiences?*; and Thread 5 - *How does a guideline to promote social and emotional well-being relate to PEH experiences?* Subsequent results drew on quantitative and qualitative findings to similarly probe experiences of outside PEH and hotel PEH to inform a data transformation into guideline-based recommendations.

#### Integrated Interpretation With Meta-analysis Thread: Guideline-based Recommendations to Inform a Health-in-All-Policies Approach to the Pandemic Response

**Fig. 3:**
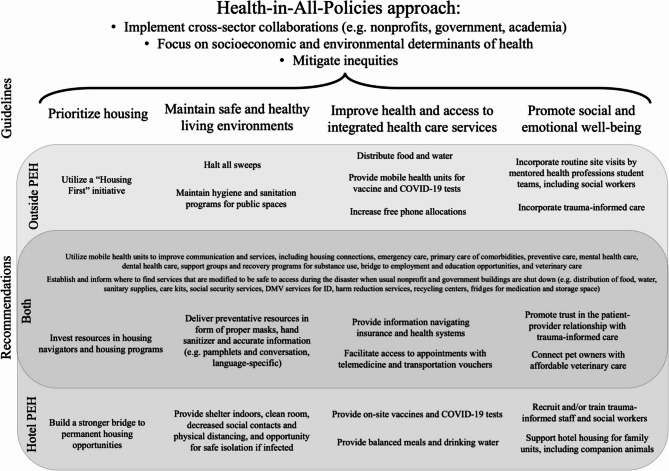
Guideline-based recommendations based on PEH and the pandemic emergency responses to inform a Health-in-All-Policies approach. This multidimensional display represents the results of the Meta-analysis Thread integrated interpretation, which involved an overlap of compare/contrast Thread 1 with guideline-based recommendations threads 2–5

Further building off the study objective, we addressed the analytic question of a Meta-analysis Thread - *How is a Health-in-All-Policies approach to future disasters informed by the impact of the disaster response on the unhoused communities surrounding Sacramento?* Thread 1 results were treated as a compare/contrast dataset, and threads 2–5 were treated as individual recommendation datasets. Datasets were iteratively interrogated to produce recommendations organized into guidelines that incorporate the comparison of the experiences of outside PEH and hotel PEH. The final guidelines included: (1) Prioritize housing; (2) Maintain safe and healthy living environments; (3) Improve health and access to integrated health care services; and (4) Promote social and emotional well-being. To maintain a comparison of similarities and differences between outside PEH and hotel PEH, the Thread 1 dataset helped to stratify recommendations. Two integrative recommendations incorporating all guidelines and both PEH populations emerged. The first of these specified the utility of mobile units for facilitating access to various services. The second incorporated the importance of modifying access to services to be safe in a pandemic. A summary of this integrated interpretation is portrayed as a multidimensional visual joint display (Fig. 3). Vaccine administration services have been added to the recommendations, as they have become available since data collection and are critical tools for the prevention of severe disease.

## Discussion

To inform Health-in-All-Policies approaches to emergency responses involving the unhoused population, this mixed methods study investigated the impact of the disaster response on PEH in a large metropolitan city in California as related to hotel room placements for prioritized at-risk PEH. An integrated interpretation placed recommendations within these disaster response guidelines to reduce morbidity and mortality during a pandemic: (1) Prioritize housing; (2) Maintain safe and healthy living environments; (3) Improve health and access to integrated health care services; and (4) Promote social and emotional well-being. The lessons learned from the pandemic emergency response may inform responses to the ongoing pandemic and other disasters, such as fires and floods. Health-in-All-Policies approaches should implement cross-sector collaborations, focus on socioeconomic and environmental determinants of health, and mitigate disparities.

In other studies, a high incidence of SARS-CoV-2 has been reported in homeless shelters, such as the study in Boston at the beginning of the pandemic [47]; and protective housing for PEH has led to a significantly lower incidence of SARS-CoV-2 positivity than the general population [48], as seen in a study in Chicago. Non-congregate hotel housing can provide safety and isolation. It can also be successfully utilized to bridge PEH to more permanent housing, as seen for our region and other states [49]. An initial goal of *Project Roomkey* in California was to provide shelter for at-risk PEH [21]. The current study showed that hotel PEH were significantly older and overall had a greater comorbidity index, suggesting that patients with more complex needs had been prioritized for emergency housing placements in hotels. Hotel placement succeeded in decreasing social contacts, increasing access to hygiene and sanitation services, and increasing access to pandemic-related preventive care such as COVID-19 testing. In addition, attitudes towards hotel rooms were overwhelmingly positive for both outside PEH and hotel PEH. However, although these efforts successfully enhanced the lives of some PEH, many PEH remain without permanent housing. As of December 2020, *Project Roomkey* in California had provided hotel rooms to more than 22,000 people; this was only about 8% of PEH in California [50].

A philosophical difference exists in approaches to care for PEH, given they are disproportionately affected by mental health, substance use disorders, and other medical comorbidities. Traditional “Treatment First” approaches emphasize treatment as a pre-requisite for transitional housing and, eventually, stable housing. Newer “Housing First” approaches drop this requirement and are based on the idea of housing as a human right [51, 52]. “Housing First” programs can integrate harm reduction approaches to reduce the negative consequences of high-risk behaviors [53, 54]. Housing is provided immediately, and there is an integration of mental health, substance use services, and attention to chronic illnesses [55]. Previous randomized controlled trials representing cities across the United States report that in comparison to abstinence-based “Treatment First” programs, “Housing First” approaches can lead to more stable housing [56–58], increased outpatient treatment [59], and fewer emergency room visits and hospitalizations [56, 60, 61]. Further, it’s been demonstrated that moving formerly unhoused individuals into supportive housing has increased access to primary care services [62]. Although not an “Housing First” program per se, the emergency hotel room placement process as part of *Project Roomkey* and the pandemic response in Sacramento did not require treatment first for PEH with substance use disorders to access a hotel room. Our work supports the ongoing literature that access to housing may be associated with increased access to various services. Employing a method to disaster response that incorporates integrated health care and “Housing First” can lead to increased access to care, hygiene and sanitation, better nutrition, decreased social contacts in a pandemic, and improved safety for a vulnerable population. Our study results align with an interview-based qualitative study from a different region in the United States, which found that benefits of hotel housing included improvements in physical health, sleep, personal hygiene, nutrition and diet, privacy, safety, and emotional well-being [63].

A Health-in-All-Policies approach includes social health. Despite being a more transient population, PEH, both living outside and in temporary hotel placements, highly valued a sense of community and social connectedness. Sanitation was often described as a community action entwined with dignity and responsibility to contribute to community wellness. Further, relationships with housed community members seemed to be a source of pride and self-confidence. Respondents living outside frequently expressed concerns that the pandemic was decreasing interaction and engagement with housed community members, and there was a preoccupation with maintaining these connections. Studies report that social factors such as limited social inclusion increase the risk of poor outcomes from the pandemic [64]. Some student volunteers were able to facilitate the use of cell phones during the response so PEH could schedule appointments and access telemedicine. More cell phone allocations during the response would improve access to care and provide an avenue for connecting with other members of society.

Community and family included both people and companion animals, as pets are often seen as family [65]. The importance of living with a pet often outweighed the option of a hotel room, as some PEH decided not to access hotels because of a limit on number of pets and/or other pet restrictions. This mirrors choices illuminated in prior hurricane studies, where some individuals chose not to evacuate because they wanted to stay with their pets [66]. The current study found that 44% of outside PEH and 29% of hotel PEH owned at least one pet. During a disaster, the vulnerability of both pet owner and pet is increased, and the loss of a companion animal can further amplify distress [66]. It has been proposed that rather than labeling pets as a risk factor to human safety during a disaster, disaster response managers can utilize animal attachment to build resilience, promote survival and facilitate recovery [67].

Cross-sector collaborations should include providers who can meet the participants where they are at. *Project Roomkey* not only provided an opportunity for PEH to access hotel housing, but it also provided an avenue where medical students who had limited access to traditional clinical placements during the initial months of the pandemic could contribute to the community in a meaningful way [21]. Our study demonstrated that a grassroots and team-based approach to the emergency response by volunteer medical students can build trust with PEH and successfully deliver needed health services to an underserved and at-risk population. Student camp leads who visited sites weekly and built rapport with PEH served as liaisons for student research team members to connect with participants interested in surveys and interviews. Prior studies have highlighted the importance of medical students in providing care for the unhoused community and reducing stigma against people who are unhoused in medical systems [68]. The current study demonstrated that many respondents benefited from student services, and a majority of respondents were interested in accessing care through a mobile health unit. Current literature supports that mobile health units can address both medical and social determinants of health, delivering services directly to vulnerable communities [69]. Mobile units can provide primary care, prevention screenings, and dental services, among other services, to a population that historically has a mistrust of the medical system. Given the large population of PEH in California, policies to support more mobile units would greatly benefit this underserved population [70].

In this study, we found that the hotels provided a safe and clean environment and access to nutritious food and clean water for PEH regardless of race, ethnicity, or income status. Individuals were triaged based on age and risk of complications from infection. Even so, not all groups had equitable access. Previous incarceration was associated with being less likely to have accessed a hotel room, suggesting a need for better integration programs after incarceration. Those who reported polysubstance use were also less likely to have accessed a hotel room. Our study found that both outside PEH and hotel PEH had negative and positive experiences with police; however, twice as many outside PEH reported negative experiences as hotel PEH. At the beginning of the pandemic, the Centers for Disease Control and Prevention issued guidelines to halt sweeps (“allow people who are living unsheltered or in encampments to remain where they are”) since displacement can increase the spread of infectious disease [46]. Not only does this guideline decrease the spread of SARS-CoV-2, but if followed, it also can support social and emotional well-being. Prior studies have highlighted the importance of trauma-informed care for PEH who have been marginalized, dehumanized, and who have endured structural violence [12]. According to the Substance Abuse and Mental Health Services Administration, the six principles of trauma-informed care include safety; trustworthiness and transparency; peer support; collaboration and mutuality; empowerment, voice, and choice; and cultural, historical, and gender issues [71]. On top of existing trauma, consequences of the pandemic can further exacerbate the ability to cope [72]. Encouraging training in trauma-informed care for all cross-sector participants in the response – including staff and volunteers, social workers, and health professions students – can help improve outcomes for those served.

A Health-in-All-Policies approach should work to mitigate disparities. The COVID-19 pandemic has exacerbated disparities based on sex and gender identity [73, 74]. Men are at higher risk of more severe disease, and it has been reported that males represent roughly 50% of Californians but 59% of total deaths due to COVID-19 in California [74]. Although domestic violence is not exclusive to one sex or gender, it is a major cause of homelessness for women [75], and lethal domestic violence incidents have increased due to the COVID-19 pandemic [76]. The current study demonstrated no difference in number of males and females accessing a hotel room (48% vs. 51%), offering shelter equitably. A previous study offered suggestions to improve the *Project Roomkey* effort, including prioritizing housing for pregnant women [77]. The COVID-19 pandemic has also exacerbated disparities based on gender identity and sexual orientation [73]. Although not explicitly asked about, sexual orientation did come up during several interviews as relating to concerns about safety. Further, our study recognizes heterogeneity in pronoun preference in the adult PEH community, highlighting the importance of including sex and gender components in studies. Previous studies have identified that up to 40% of youth PEH identify as LGBTQ+, and > 55% of LGBTQ + adults live in poverty in California [73]. As this study was limited to adults 18 years and older and did not explicitly ask about sexual orientation, future studies should include the experiences of unhoused youth and provide an option to share experiences relating to sexual orientation. Regardless of identity, the most mentioned priority of participants was housing, and stronger bridges to more permanent housing are needed, independent of a pandemic.

A significant limitation of the quantitative portion was that many hotel PEH may not have originated from one of the survey areas for the outside PEH. Many of these individuals may have come directly from a hospital [50]. This would underestimate the access to student services in the field. It is important to consider that due to the nature of the rapid pandemic response, it is highly likely that additional confounding factors not described here could contribute to inherent differences between hotel PEH and outside PEH population. Hotel placements were triaged initially by older age and comorbid conditions; as such, statistically significant differences in group comparisons are more of a reflection that planned prioritization and protective public health measures had been carried out robustly. Furthermore, we could not infer causality due to the cross-sectional design of the data collection. There may be recall bias that increased primary care physician visits for those in housing, but previous work has demonstrated a “Housing First” approach has been associated with decreased emergency room visits and increased outpatient care in randomized controlled trials [52, 59]. In addition, this study did not account for many variables – having a working phone, access to transportation, employment status, and duration of homelessness – which may have impacted a respondent’s ability to both obtain hotel placement and primary care physician access and thus confounded the results. Furthermore, this study relied on self-reported data; however, we did not expect these answers to differ among the two study arms. This study may have limited generalizability to PEH in other states since California has a significant unsheltered population [78]. However, the focus on a Health-in-All-Policies approach may yield parallel solutions in different locations.

Due to the momentum of the pandemic response, some qualitative interviews led to very short answer responses or inadequate recording quality, and related transcripts were less appropriate for an in-depth coded analysis. To address this, we coded and analyzed the most substantive transcripts that came from recordings with minimal background noise. Of the original twenty-five outside PEH and forty-seven hotel PEH interviews, nineteen and sixteen, respectively, were included in full analyses. We do not expect the study’s overall findings to be significantly altered by the exclusion of low-quality transcripts. Granted, a limitation of the qualitative interviews portion is potential selection bias.

A benefit of using both quantitative and qualitative approaches is simultaneously assessing problem magnitude and meaning [79]. Then, combining interpretations of quantitative, qualitative, and integrated approaches can bring a more holistic analysis to a complex dataset [42]. However, a limitation of mixed methods research is that a definition of integration is not widely agreed upon; therefore, we made sure to specify the approach that guided our analyses and interpretations. In addition, a limitation of the overall study is that mainly English-speaking adults were included. Follow-up studies should collaborate with language services to incorporate accurate and culturally competent data collection, analysis, and interpretation for Sacramento’s non-English speaking and hearing-impaired PEH.

## Conclusion

By using a student-powered mixed methods approach to public health research, we explored the impact of the disaster response on the unhoused population in Sacramento. Our integrated interpretation illuminated the importance of *Project Roomkey* emergency hotel room placements in relation to physical health and the social, economic, and environmental determinants of health. Combining findings, we synthesized recommendations to inform a Health-in-All-Policies approach to the emergency response that included protection from infectious disease and health improvement from non-communicable disease. The next steps in a larger vision of team science include a translation phase [80], where leaders spearheading cross-sector collaborations apply these findings to practice to improve outcomes for the unhoused population during the response to a disaster, such as the ongoing pandemic as highly transmissible variants emerge, or other future disasters.

## Supplementary Information

Additional file 1. Overview of team-based approach

Additional file 2. Survey questions

Additional file 3. Modified comorbidity index

Additional file 4. Interview questions

Additional file 5. Self-reported sex, gender and pronoun diversity

Additional file 6. Final codebook from qualitative analysis

Additional file 7. Examples of interview segments categorized by subtheme

Additional file 8. Summary of representative quotes in the main text by theme

Additional file 9. Number of interview transcript analyses involving the use of each code

Additional file 10. Interview responses to the question: What is your most important need now?

## Data Availability

The datasets used and/or analyzed during the current study are available from the corresponding author.

## Abbreviations

COVID-19: Coronavirus disease 2019
PEH: Person/people experiencing homelessness
IRB: Institutional Review Board
COREQ: Consolidated Criteria for Reporting Qualitative Research
AAPOR: American Association for Public Opinion Research
STROBE: Strengthening the Reporting of Observational Studies in Epidemiology
IRR: Incidence Rate Ratio
REDCap: Research Electronic Data Capture
CI: Confidence Interval
aOR: adjusted Odds Ratio
