# Supplementary material for: COVID-19 response and the unhoused communities in Sacramento: a mixed methods study with policy implications
Source: BMC Public Health. 2025 Nov 18;25:4012. doi: 10.1186/s12889-025-24515-0 (PMC12625094; doi:10.1186/s12889-025-24515-0)
Supplement: Supplementary file 1 — Additional file 1. Overview of team-based approach [file 12889_2025_24515_MOESM1_ESM.pdf]

Additional file 1: Overview of team-based approach

| **Student team** | Field research | Remote research | Quantitative | Qualitative coding | Qualitative theme analysis | Policy | Literature review | Mixed methods project conception, design and management |
|---|---|---|---|---|---|---|---|---|
| **# of researchers** | 14 | 21 | 11 | 14 | 8 | 10 | 10 | 3 |

As related to Figure 1. Researchers were able to serve on more than one team.
