# Supplementary material for: COVID-19 response and the unhoused communities in Sacramento: a mixed methods study with policy implications
Source: BMC Public Health. 2025 Nov 18;25:4012. doi: 10.1186/s12889-025-24515-0 (PMC12625094; doi:10.1186/s12889-025-24515-0)
Supplement: Supplementary file 2 — Additional file 2. Survey questions [file 12889_2025_24515_MOESM2_ESM.pdf]

Additional file 2: Survey questions

Field Site:	Team Member:		Date (ex. 05/31/2020): / /
Unique ID:		Primary language:	
Age:		Race/Ethnicity (circle): (Non-Latino) White<br>Hispanic/Latino (Non-Latino) Black<br>(Non-Latino) Asian Mixed Other	
Sex:			
Gender:			
Pronouns:		Height (self report, ft/in):	
Veteran (circle)? Yes No Unk		Weight (self report, pounds):	

Access to services

What services from us have you been able to use (circle all that apply)?

Hand-washing station Wound care Mask Hand sanitizer/soap COVID-19 test
Info on COVID Food Street clinic Telemedicine Link to housing Other:

	Circle answer		
Do you have a primary care provider?	Yes	No	Unk
- **If YES,** how many times in the last month have you seen your primary care provider (#)?			
Do you have an insurance plan? **If YES,** what?	Yes	No	Unk
Do you have access to working hand-washing stations?	Yes	No	Unk
Do you have access to a bathroom?	Yes	No	Unk
Do you use hand sanitizer? **If YES,** when?	Yes	No	Unk
Do you use to a face mask? **If YES,** when?	Yes	No	Unk
Do you have access to a shower?	Yes	No	Unk
If you could, would you get into a hotel room / would you stay in a hotel room?	Yes	No	Unk
If we were able to provide a mobile clinic, would you use its services?	Yes	No	Unk

**COVID-19 testing results (self-reported):**			
Have you had a COVID19 test? **If YES:** date of test: / /	Positive	Negative	Undetermined
Do you know someone who has COVID-19?	Yes	No	Unk
- **If YES,** in what capacity?			
- Any chance you were exposed? **If YES,** date of exposure: / /	Yes	No	Unk
**Specific social history**			
How long have you been living where you are currently?			
How many times have you relocated your sleeping location in the past month (#)?			
When did you last stay in a shelter?			
How many people have you had close contact to in the last 24 hours?			
Do you have pets?	Yes	No	Unk
- **If YES**, how many and what species?			
- **If YES**, where do your pets sleep?			
How many people do you live with?	# adults: # children:		
Where do you sleep?			
How many people sleep within 10 feet of you (#)?			
How many meals per day do you eat (#)?			
Where do you get your meals?			
What did you have for dinner last night? Circle which categories are included:	protein	vegetable	grain/carbs
Where do you get water?			
Do you have access to electricity? **If YES,** describe:	Yes	No	Unk

**Do you have any of the following conditions?**			
Lung disease (asthma)	Yes	No	Unk
Lung disease (COPD/emphysema)	Yes	No	Unk
Other lung disease; **If YES,** specify:	Yes	No	Unk
Diabetes mellitus	Yes	No	Unk
Hypertension	Yes	No	Unk
Heart disease	Yes	No	Unk
Kidney disease	Yes	No	Unk
Liver disease	Yes	No	Unk
Cancer; **If YES,** specify:	Yes	No	Unk
HIV positive	Yes	No	Unk
Other immunocompromised condition; **If YES,** specify:	Yes	No	Unk
Neurologic/neurodevelopmental; **If YES,** specify:	Yes	No	Unk
Other chronic diseases; **If YES,** specify:	Yes	No	Unk
Mental illness; **If YES,** specify:	Yes	No	Unk
Are you prescribed medication?; **If YES,** how many medications?	Yes	No	Unk
If prescribed, do you currently take your medication?	Yes	No	Unk
**General social history**			
Current smoker (cigarettes); **If YES,** how many packs/wk?	Yes	No	Unk
Former smoker (cigarettes); **If YES**, how many packs/wk?	Yes	No	Unk
Marijuana use; **If YES**, what route?	Yes	No	Unk
IV drug use (heroine)	Yes	No	Unk
Meth use; **If YES**, what route?	Yes	No	Unk

Alcohol consumption; **If YES**, how many drinks/wk?						Yes	No	Unk
Recent incarceration; **If YES**, for how long? Date of release: / /						Yes	No	Unk
If applicable, currently pregnant					N/A	Yes	No	Unk

*As part of Disaster Service Worker volunteer service, a symptom survey was also administered to connect individuals with testing and services. Results not reported here.
