# Supplementary material for: COVID-19 response and the unhoused communities in Sacramento: a mixed methods study with policy implications
Source: BMC Public Health. 2025 Nov 18;25:4012. doi: 10.1186/s12889-025-24515-0 (PMC12625094; doi:10.1186/s12889-025-24515-0)
Supplement: Supplementary file 3 — Additional file 3. Modified comorbidity index [file 12889_2025_24515_MOESM3_ESM.pdf]

Additional file 3: Modified comorbidity index

Modified Comorbidity Index		
	Score Assigned[1]	Comorbidities Reported in Study Population
Myocardial Infarction	+1	N/A
Congestive Heart Failure	+1	N/A
Peripheral Vascular Disease	+1	N/A
Cerebrovascular Disease	+1	Stroke
Dementia	+1	N/A
Chronic pulmonary disease	+1	Chronic Obstructive Pulmonary Disease, Chronic Bronchitis
Rheumatologic disease	+1	Rheumatoid Arthritis,<br>Ehlers-Danlos Syndrome
Peptic Ulcer Disease	+1	N/A
Liver Disease	+1	Other[2]
Diabetes[3]	+1	Diabetes Mellitus
Hemiplegia or paraplegia	+2	N/A
Renal Disease	+2	Other[2]
Any malignancy, including lymphoma and leukemia[3]	+2	Brain Cancer, Esophageal Cancer, Lung Cancer, Breast Cancer, Ovarian Cancer, Liver Cancer, Prostate Cancer, Lymphoma
HIV	+3	HIV+

[1] Scores assigned using the Deyo 1987 and Romano 1987 adaptations of the Charlson Comorbidity Index
[2] Specific comorbidities not collected, see survey.
[3] Severity/stage/location not collected, used lowest score category
[4] Used score +3 in accordance to Glasheen WP, Cordier T, Gumpina R, Haugh G, Davis J, Renda A. Charlson Comorbidity Index: *ICD-9 Update* and *ICD-10* Translation. *Am Health Drug Benefits.* 2019;12(4):188-197.
