# Supplementary material for: COVID-19 response and the unhoused communities in Sacramento: a mixed methods study with policy implications
Source: BMC Public Health. 2025 Nov 18;25:4012. doi: 10.1186/s12889-025-24515-0 (PMC12625094; doi:10.1186/s12889-025-24515-0)
Supplement: Supplementary file 4 — Additional file 4. Interview questions [file 12889_2025_24515_MOESM4_ESM.pdf]

Additional file 4: Interview questions

Qualitative Assessment (outside PEH):
Experiences and Perceptions during the COVID-19 Pandemic in the Unhoused Community
Answers to be audio recorded. Script as follows:

If you agree to take part in the research and allow the interview to be recorded, I ask for your verbal consent. Do you fully understand that this will be recorded? Do you consent to being recorded?

The date is [date]. Camp code is [camp code]. Time of collection is [time]. Unique ID is [unique ID same as survey].

We were hoping to get a sense of how are you experiencing the COVID-19 pandemic.

[1.] Please describe what you've heard about the COVID-19 infection.
- How did you first hear about it?
- Who do you trust to provide information about COVID-19?
- Where did it come from?
- What causes COVID-19?
- How does one get COVID-19?
- What can COVID-19 do to you?

[2.] Please describe if you've known anyone with the COVID-19 infection.
- How concerned are you about catching or spreading it?
- If you found out you tested positive but didn't have symptoms, what do you think you would do?
- What do you think about wearing a face mask?
- What sanitation sites (like toilets and handwashing stations) do you have access to?
  - How has access to these sanitation sites affected you?

[3.] Please describe if you have been able to access COVID-19 testing and how this has affected you.
- What do you see as the barriers to COVID-19 testing in your community?

[4.] How has your life changed since COVID-19?
- How has your access to health care changed since COVID-19?
- How has your access to harm reduction changed since COVID-19?
- How has your access to food and water changed since COVID-19?

[5.] Depending on the site, our student group has been able to provide hand-washing stations, wound care, masks, hand sanitizer/soap, food, COVID-19 information, COVID-19 testing, a street clinic, telemedicine visits, and a link to hotel housing. Of these services, what have you been able to access?
- What was the most important to you and why?
- What services do you still need and why?

[6.] What are the barriers to seeking health care?

[7.] Please describe if you are aware of hotel rooms becoming available and what you think of this process.

[8.] Please describe if there have been any police sweeps recently and how they have affected you.

[9]. Please describe if you have a pet and what role your pet plays in your life.

[10.] Please describe how you identify in terms of your housing status (for example: unhoused, homeless, I have a home, something else) and why.

[11.] In your life right now, what is your biggest fear?

[12.] In your life right now, what is your most important need?

Qualitative Assessment (hotel PEH):
Experiences and Perceptions during the COVID-19 Pandemic in the Unhoused Community
Answers to be audio recorded. Script as follows:

If you agree to take part in the research and allow the interview to be recorded, I ask for your verbal consent. Do you fully understand that this will be recorded? Do you consent to being recorded?

The date is [date]. Camp code is [camp code]. Time of collection is [time]. Unique ID is [unique ID same as survey].

We were hoping to get a sense of how are you experiencing the COVID-19 pandemic.

[1.] Please describe what you've heard about the COVID-19 infection.

- How did you first hear about it?
- Who do you trust to provide information about COVID-19?
- Where did it come from?
- What causes COVID-19?
- How does one get COVID-19?
- What can COVID-19 do to you?

[2.] Please describe if you've known anyone with the COVID-19 infection.

- How concerned are you about catching or spreading it?
- If you found out you tested positive but didn't have symptoms, what do you think you would do?
- What do you think about wearing a face mask?
- What sanitation sites (like toilets and handwashing stations) did you have access to prior to the hotel?
    - How did access to these sanitation sites affect you?

[3.] Please describe if you have been able to access COVID-19 testing and how this has affected you.

- What do you see as the barriers to COVID-19 testing in your community?

[4.] How has your life changed since COVID-19?

- How has your access to health care changed since COVID-19?
- How has your access to harm reduction changed since COVID-19?
- How has your access to food and water changed since COVID-19?

[5.] Depending on the site, our student group has been able to provide hand-washing stations, wound care, masks, hand sanitizer/soap, food, COVID-19 information, COVID-19 testing, a street clinic, telemedicine visits, and a link to hotel housing. Of these services, what have you been able to access?

- What was the most important to you and why?
- What services do you still need and why?

[6.] What are the barriers to seeking health care?

[7.] Please describe your process of accessing a hotel room during the pandemic.

- What do you think of this process?
- What services have you been able to access while staying in a hotel room?
- What do you like about the hotel situation?
- What would you change about the hotel situation?
- What are your plans for after staying in the hotel?

[8.] Please describe if there have been any police sweeps (prior to the hotels) recently & how they have affected you.

[9]. Please describe if you have a pet and what role your pet plays in your life.

[10.] Please describe how you identify in terms of your housing status (for example: unhoused, homeless, I have a home, something else) and why.

[11.] In your life right now, what is your biggest fear?

[12.] In your life right now, what is your most important need?
