# Supplementary material for: COVID-19 response and the unhoused communities in Sacramento: a mixed methods study with policy implications
Source: BMC Public Health. 2025 Nov 18;25:4012. doi: 10.1186/s12889-025-24515-0 (PMC12625094; doi:10.1186/s12889-025-24515-0)
Supplement: Supplementary file 5 — Additional file 5. Self-reported sex, gender and pronoun diversity [file 12889_2025_24515_MOESM5_ESM.pdf]

Additional file 5: Self-reported sex, gender and pronoun diversity

Sex = Male, gender ≠ "male"	• 1 x gender = other, pronoun = they<br>• 2 x gender = unknown, pronoun = he
Sex = Female, gender ≠ "female"	• 3 x gender = unknown<br>○ 2 x pronoun = unlisted other pronouns<br>○ 1 x pronoun = unknown

10 x Gender = Male, pronoun ≠ "he"	2 x unlisted pronouns,<br>1 x no preference,<br>7 x unknown
13 x Gender = Female, pronoun ≠ "she"	5 x unlisted pronouns,<br>1 x no preference,<br>6 x unknown<br><br>1 x unlisted & unknown
1 x Gender = Something else	1 x they
5 x Gender = Unknown	3 female (pronouns = 2 unlisted, 1 unknown)<br>2 male (pronouns = 2 x "he")
