# Supplementary material for: COVID-19 response and the unhoused communities in Sacramento: a mixed methods study with policy implications
Source: BMC Public Health. 2025 Nov 18;25:4012. doi: 10.1186/s12889-025-24515-0 (PMC12625094; doi:10.1186/s12889-025-24515-0)
Supplement: Supplementary file 6 — Additional file 6. Final codebook from qualitative analysis [file 12889_2025_24515_MOESM6_ESM.pdf]

Additional file 6: Final codebook from qualitative analysis

Code Category	Code	Brief Description	Additional Criteria	Examples
any	ACCESS	discussion of difficulty or ability to access beneficial resource		ex. under SUBSTANCE, interviewee discusses ACCESS to detox
any	CAUSE	interviewee identifies cause of situation being faced		cannot find housing, CAUSE - bad credit
any	CHANGE	a typical situation is different than it used to be or lack of different situation. usually about ACCESS		NO CHANGE means absence of change, YES CHANGE means change happened
any	COPING	interviewee indicates that they see what they are discussing as coping, managing negative experiences, recovering from negative experiences		
any	FEAR	specific fear of topic/issues or lack thereof		
any	FUTURE	indicate future planning/ goal-setting, or acknowledgement of lack thereof, feelings of hope or despair		

any	HUMANITY	social responsibility, concerns on spreading virus to others, personal action plan if tested positive, compassion towards others, esp. about society as a whole		
any	IDENTITY	how a person identifies themself, matters of personal identity. housing status as identity?		ex. identity of housing status
any	INDIFF	interviewee indicates lack of interest/value in a topic, issue, resource		
any	INFO	interviewee professes information about disease, consequences, health risks, also other information that are unrelated to health. also discusses source of information or lack of information or desire for information.	modifiers used: TRUE (for accurate INFO) FALSE (for inaccurate INFO), NO (for lack of INFO such as "I don't know") and YES (if someone just says I have information)	
any	NEED	self-identification of a need		
any	PRIORITY	interviewee indicates that this is their primary priority		
any	RESPECT	discuss experiencing dignity or indignity from others		
any	SLICE OF LIFE	any discussion of activities, ideas, conversation that is not related to other codes but about human interests, could be mundane	useful for showing "regular" human interests and functioning of folks who are otherwise othered by society	

any	SOLUTION	interviewee mentions a successful or unsuccessful social intervention or a proposed intervention, including what the interviewee proposes		
any	SPIRITUALITY	any discussion of faith, religion, spirituality or naming the lack thereof		
any	STRESS	interviewee indicates some level of worry or anxiety or stress arising from situation/need	stress can be anxiety about something that has or has not occurred, or something that causes stress	
any	TEAM	any discussion of the involvement or actions or effects or lack thereof of our Encampment Med Team		
any	TECHNOLOGY	discuss technology as tools (for survival or barrier)		
any	TRANSPARENCY	interviewee indicates clarity or lack thereof of resources or leadership, guidance from authority, access to quality/reliable information. may include frustration/confusion over how to access resource		
any	TRUST	interviewee indicates trust or distrust in provider, service, system		
any	UNCERTAINTY	unsure about a type of knowledge or indecision for actions		

BASIC NEEDS	CLOTHING	discussion of clothing as a resource		
BASIC NEEDS	FOOD	discussion of food		
BASIC NEEDS	ID CARD	any discussion of the resource or absence of ID documents		
BASIC NEEDS	MONEY	any discussion of personal finances, jobs, lack of job, and generally finances as a resource	welfare check is an example of personal finances that comes from government, this code is not about government funds in the general sense, this is specifically about how it relates to personal finances or job	
BASIC NEEDS	NETWORK	comments on networks, relationships, family, relatives, friends		
BASIC NEEDS	PET	discussion of companion animal(s)		
BASIC NEEDS	SAFETY	indication of feelings of safety, insecurity, danger, security, etc.		ex. feelings after having a negative COVID-19 test: "well it made me feel better. that means I'm not gonna catch it, or I didn't have it."

BASIC NEEDS	SANITATION	comments on environmental health, trash build-up, use, water quality (non-drinking)		
BASIC NEEDS	SHELTER	any discussion of need or use or lack of shelter, such as protection from the elements: sleeping bag, use of cooling centers, library		
BASIC NEEDS	TRANSPO	discussion of transportation		
BASIC NEEDS	WATER	drinking water, hydration, or dehydration		
HEALTH	BENEFIT	indicates that subject/situation resulted in health benefit		
HEALTH	COMMUNITY-RISK	self-perceived probability of harm to community, consider size of harm as well as likelihood		
HEALTH	COVID	view on COVID-19, perceptions, reflections, opinions		
HEALTH	DISABILITY	discuss disability status and/or resources for disability		
HEALTH	HARM	indicates that subject/situation resulted in health harm		

HEALTH	HARM REDUCTION	discussion of services including harm reduction, detox, etc.		
HEALTH	HEALTHCARE	discuss interaction / perspective with healthcare		
HEALTH	IMMUNITY	describe status or effective/ineffectiveness of immune system, health status		
HEALTH	MANAGEMENT	discussion of ability to or actions involved in managing health		
HEALTH	MENTAL	discussion of mental health, management of own diagnosis, prescriptions, access		
HEALTH	OTHER	discuss own health in another way (if not captured by any other specified HEALTH code)		
HEALTH	PREVENT	discussion of preventative care		
HEALTH	REPRO	discussion of women's/reproductive care		
HEALTH	SELF-RISK	self-perceived probability of harm, consider size of harm as well as likelihood		
HEALTH	SURGERY	discussion of surgery		

HEALTH	TESTING	discussion of COVID-19 testing		
HEALTH	TRAUMA	interviewee indicates significant event/experience of trauma	an event, series of events, or set of circumstances that is experienced by an individual as physically or emotionally harmful or threatening and that has lasting adverse effects on the individual's functioning and physical, social, emotional, or spiritual well-being.* trauma must have occurred, cannot be an anxiety about a potential fear	
HOUSING	DISPLACEMENT	discuss relocation or need to relocate, loss of housing		
HOUSING	HOUSE	discussion of housing situation		living outside, at a friend's, in a car, etc.
HOUSING	STABILITY	any discussion of the effects of housing (in)stability and feelings that arise from it		
POWER	EXTERNAL	express belief that external power has agency, could be God or government		
POWER	INPUT	interviewee discusses opportunities for or lack of input, or desire to give input in decisions that affect their life		

POWER	SELF	express belief that interviewee has agency		
PUBLIC SERVICES	CRIME	any discussion of acts of crime or fear of or lack of crime aka violence or theft or intimidation, not necessarily involving police		
PUBLIC SERVICES	GOVERNMENT	any discussion of the role of government or actions taken by government or lack thereof		
PUBLIC SERVICES	INCARCERATION	any discussion of incarceration, whether it be fear of, lack of, or experience of		
PUBLIC SERVICES	LEGAL	any discussion of engagement with the legal system, challenges wherein, lawsuits, legal basis for an action, lack of legal basis, etc.		
PUBLIC SERVICES	POLICE	interaction with law enforcement		
SUBSTANCE	METH	any mention of methamphetamine specifically		
SUBSTANCE	OTHERSUB	mentions usage or perspective of substances		

*Definition of trauma from: Substance Abuse and Mental Health Services Administration (SAMHSA). Trauma-Informed Care in Behavioral Health Services. Treatment Improvement Protocol (TIP) Series 57. HHS Publication No. (SMA) 13-4801. (Available at: https://www.ncbi.nlm.nih.gov/books/NBK207201/)

Code Category	Code	Brief Description	Additional Criteria	Examples
quote	GREAT	good for quoting in article, concisely expresses major theme in a new or powerful way		
quote	REC	a quote that could directly inform a policy recommendation		
quote	UNIQUE	unique response to a given question		
modifier	IN	discussion of someone who is inside of a subject's chosen network, people that they associate with	modifier for NETWORK	
modifier	OUT	discussion of someone who is outside of subject's chosen network, a stranger, or people they don't want to associate with	modifier for NETWORK	
modifier	POS	positive experience or opinion		
modifier	NEG	negative experience or opinion		
modifier	YES	presence of subject that is being modified		YES ACCESS means presence of access

modifier	NO	absence or lack of subject that is being modified		NO INFO means lack of info such as "I don't know"
modifier	STRONG	high level belief or feeling		ex. misinformation: “aliens from outer space made the virus”
modifier	MOD	moderate level belief or feeling		
modifier	WEAK	low level belief or feeling		ex. misinformation: “I wish we didn’t have to wear masks inside”
modifier	TRUE	accurate. researcher's perception of accuracy of INFO statement		
modifier	FALSE	inaccurate, researcher's perception of inaccuracy of INFO statement		
