# Supplementary material for: COVID-19 response and the unhoused communities in Sacramento: a mixed methods study with policy implications
Source: BMC Public Health. 2025 Nov 18;25:4012. doi: 10.1186/s12889-025-24515-0 (PMC12625094; doi:10.1186/s12889-025-24515-0)
Supplement: Supplementary file 7 — Additional file 7. Examples of interview segments categorized by subtheme [file 12889_2025_24515_MOESM7_ESM.pdf]

Additional file 7: Examples of interview segments categorized by subtheme

Subthemes	Quotes Relating to Outside PEH	Quotes Relating to Hotel PEH
Information	[Researcher: What are the barriers to seeking health care?] Probably the ability to know what resources are available to us. And how to obtain them with everything. It's kind of difficult to know what services.<br><br>I got a text from uh, a lady friend, she did a chain text, and she said, 'Hold your breath 10 seconds in the morning, and if you don't start coughing real bad after 10 seconds of holding your breath in this area, then you don't have it.' And I been doin' that ever since I read that.<br><br>[Researcher: Please describe what you've heard about the COVID-19 infection.] It's really killed a lot, quite a few people. Airborne and affects mostly the elderly, but that's not true, it affects a lot of different ages. And do not share drinks or smokes or anything with anybody. Stay 6 feet away from everybody. Try to not share any drinks or anything like that. [Researcher: How did you first hear about it?] On the news. [Researcher: Who do you trust to provide information about COVID-19?] I trust the news. [Researcher: Do you know where the virus came from?] They say it came from China, but I don't really know. [Researcher: Do you know what causes COVID-19? How does the virus spread?] Airborne, and you touch it, and sharing drinks, sharing - I don't really know, but what I heard just from sharing anything. Even touching something, and then touching yourself or your face or your mouth. [Researcher: What can COVID-19 do to you?] Kill you.<br><br>[Researcher: How did you first hear about it?] That it's deadly, that's about it. [Heard about it] on the news [gets access to the news] from my auntie. [Researcher: did anyone else talk to you about it?] People that come around and visit -bring water and shit like that. [Researcher: Who do you trust to provide information about COVID-19?] Nobody. I don't know, I don't know if it's real or not, I don't know. [Researcher: Where did it come from?] Who knows, I don't know where it came from – China? [Researcher: What causes COVID-19?] Germs? I don't know what causes it. I have no idea. [Researcher: Where do you normally trust medical information from?] I don't trust at all, and I don't know if it's real or not. I mean I don't know anyone that caught it yet.	It's hard to get any information. I've got more information since we've been here [at the hotel] and I've watched the news. The homeless can't just watch the news. Some have a radio, but most don't.<br><br>[Researcher: What services do you still need and why?] Services, I need…the government office to be open. [Researcher: For jobs?] DMV. –With the situation going on, everything expired. You can't do nothing. So it takes longer. Now you don't have a driver's license, you need an ID… you need [your] birth certificate…and it just becomes hard. Those things are real necessary. And it's not like you don't use them all the time, but when you need them now, you really need them and then you have no access to them…the office is not open.<br><br>I actually almost wanted to believe it was a hoax, but they [both "high society" and "low society" people who did not think it was a hoax] I couldn't ignore. I washed my hands tons and tons all the time before I got in the street. I social distanced to the point where I would tell people "Excuse me you're in my area." And that kept me negative. And that's why, because I was a little more aggressive than the average person who probably didn't believe at first. Especially when you guys said don't wear masks. Like, when President Trump said "Don't wear masks and leave the masks to the workers because there's a shortage." And then people were like "If we don't need masks and if only nurses need it, then it's bull, it's a hoax," which is true. I knew that that was wrong. Just like when AIDS came out and they said through needles, through sex, and then they tried to say that but not oral sex. "You can't get it through oral sex, either way." And I just knew that was bullshit. And just three years, I was right. It was bullshit.<br><br>[Researcher: Please describe if you have been able to access COVID-19 testing and how this has affected you.] Actually no, that was so hard to get. I went to the hospital even just to check, you know, right when I found out about it [COVID-19]. They didn't even have testing. Um, they…they listed off a couple places that had free testing but …I was walking everywhere at the time so I wasn't going to walk just all the way back but…I mean it [became] easy when [we] came here [to the hotels].
Sanitation	[Researcher: Have you seen any changes in access to hand washing stations or bathrooms because of COVID?] You can't get access. You can't use the public bathrooms anymore.<br><br>[Researcher: What is your knowledge about how the virus works?] I think the main thing is that it's spread through social contact. Being in someone's face and just standing under 6 feet. I mean, the breath and the saliva and the spit of a person	[Researcher: How do you maintain your rooms here?] Well they want us to be independent and to use our own resources to be able to do those things. But you can ask them for very, very basic. They have Clorox and a broom that the teeths are only half. That's it. And you can get towels. You exchange your own towels for new ones. But I visited a few people, and they're still living homeless, like they're homeless. Not very clean, doesn't smell very clean. Um, not all but some. And I don't think they're doing it on purpose. I think they've been living like this

	goes at least a couple of feet. I mean and it goes beyond someone standing. There are germs and stuff in the air right now, we just can't see them. And now it's finally caught up with us. And we were dirty, we were not being clean enough. C'mon that's what this pandemic is all about. It's clean-li-ness. Homeless people can't be washing their hands every 20 minutes. It's impossible. And social distancing? It's impossible.<br><br>We are considered the lost, the not worthy of anything in life...we are treated as garbage. I have physical conditions that make it hard for me to even be able to use the restroom with some form of dignity and then feel like there is some kind of sanitation.<br><br>[Researcher: What sanitation sites did you have access to?] Well toilets was very hard to come by downtown especially when the pandemic started. But we had the hand stations at City Hall, hand washing stations. And during the day we had restrooms at City Hall and at Capitol Park. But, weekends and late afternoons, bathrooms were kind of hard to come by. [Researcher: So the entire weekend you had no access?] Pretty much, unless we were at the park and they were open until 3 or 4 and then they close them down. So you have all these people downtown, homeless with no restroom. And we kept hearing that they were gonna put washing stations in the park across the street. Which they did, but they didn't maintain them. We were just kind of lost down there.	for so long. There needs to be classes to make it more successful for a homeless person to go back to society and be regular. You need a course, you need to do a free course for them to learn. I don't think that they realize – I've seen it, I've seen it some many times – and then they lose their homes. Nobody wants to deal with that. These are grown people, they shouldn't have to deal. Teach them, hygiene, how to clean a bathroom. Basic shit that you should know already. And it's not that they don't know, it's just after a while, you just don't give a fuck. You don't believe anymore.<br><br>[Researcher: How strict are they about rules here (at the hotel)?] This program, they're very strict on rules…you know there's certain rules – like if you were a resident here or so long as I'm a resident, I can't go in your room and you can't go in my room. It's a good rule, and that's a major rule. Not breaking curfew. We're not allowed to smoke in the very front because they want people on site, so you gotta smoke in the back. What else is there… you can't threaten other residents. You can't threaten to hurt yourself or hurt others. You're not supposed to share cigarettes – you can do it if you're boyfriend/girlfriend, husband/wife, they don't seem to have a problem with that – but they don't like you to share cigarettes with three people or something like that because they're worried about the virus.
Sanitation as a Community Action	For a while, I got known to where, you know, I've had park rangers give me trash bags 'cause they'd see me picking up trash and now if every homeless person did that, we wouldn't get fines...They'd see that eh- at least we're, you know, doing a good job keeping everything clean.<br><br>[Researcher: So you were able to construct your own handwashing stations?] Yeah. What we did was we went to Walmart, and we got a 5 gallon water bottle and a pump that has a sprayer on it. And we got anti, we bought up all the anti-bacterial dishwashing, and we got bleach, put 4 packs of bleach in it, so we had anti-bacterial dishwashing soap and in it had bleach, and we were keeping our hands and anything we drank out of clean. In my group, we made a strict no canning rule, no one was allowed to go canning anymore. Cuz we didn't want anybody touching anything that anybody was putting their mouths or anything on, so that's what we did. Well, it's my camp. And then I have another married couple who stays with me in there, who lives in there with me. So they're my age, and he has respiratory problems, so…And then there's a young kid in there, that was with us, so we just made the rule nobody's allowed to go get cans any longer. And we made up our handwashing stations, so…<br><br>[Researcher: What do you think about wearing a face mask?] Very courteous and wonderful thing to do as long as you have one. As long as you have access to one.	[Researcher: Please describe if prior to the hotel, if there were any police sweeps in your area and how that affected you.] Daily. At city hall we fought them and we were actually in the beat. They did a sweep in the morning once this all started, the pandemic, and they weren't supposed to be doing sweeps, but they continued to clear us out every morning at 6am because it was city hall and they were still open. They kind of butted heads there. Crystal at the Homeless Union, you know Crystal? She's a sweetheart. She said 'You guys just stick to your guns, take your time, I have the beat coming out.' Except on weekends because we could stay at City Hall on weekends.<br><br>[Researcher: What do you think about wearing a face mask?] Oh, it's fine by me… if it keeps anyone else safer.<br><br>[Researcher: What role does wearing a facemask play?] It's a big role. Going out in public with other people, I wear it.<br><br>[Researcher: How concerned are you about catching or spreading the virus?] Well I'm pretty concerned catching it. I don't wanna catch it because I'm already smoking cigarettes. So It's like yeah… so it's like I think if you care for yourself you care for others. When you don't care for yourself, a lot of things in life you know like. Whatever can happen to you can happen to me. If im sick and I don't

	A lot of us here do not have access to one every few days at least. A lot of us here do not have access or money to keep face masks. Even the ones that are given out that are washable we don't have the money to wash our clothes anyways.	take care of myself you can get sick. So it's like people have to love themselves so like you like they can love everybody else. Or something. Or not love but like care not to get them sick.
Trust with Providers	I don't really talk to them [unfamiliar outreach workers]. I talk to you guys [volunteer medical students].<br><br>[Researcher: Our student group has been able to provide different services. Of these, what was the most important to you and why?] What was most important. What hit my heart? The nurses [the female medical students on our team were often mistaken as nurses] on their time off would come around to the homeless people, on their own time, and offer hand sanitizer and ask you what's wrong with you if you need any help. Do you need any suggestions on where to go what to do. That has probably meant the most.<br><br>[Researcher: Who do you trust to provide information about COVID-19?] My doctors and the students that come out and help us.	[Researcher: Please describe if you have been able to access COVID-19 testing and how this has affected you.] Oh yeah if I ask for it, I guess they give to you. I dunno if they give it to you, I haven't asked for that. But they check your vitals, they come and check your heart rate. They got registered nurses who come and observe how you doing, they say: "you look a little out of breath." Like they know and care. So it's up to you if you wanna go to doctors or not. But I'm feeling okay but I know… I think if I ask them, they're do whatever they do to get it done.<br><br>[Researcher: Please describe your process of accessing a hotel room during the pandemic.] The process, he was askin' about the process too, the process it just went from bein' in the hospital and the lady hearin' my story and she felt empathetic she-she made- she made calls like she said she was gonna do. She said she couldn't promise anything, but she made calls, and everything she was gonna- everything she was tryin to do she did. Yep. Brought a tear to my eye [laughs].
Chronic Health Conditions	[Researcher: We were hoping to get a sense of how you are experiencing the COVID-19 pandemic.] For me, it affected me because I can't get to my primary care doctor. 'Cause they're- they're even refusing to answer the phone. I've left messages, they're not returning calls and I have a lot of medical issues. Um, I'm diabetic, I'm epileptic, and I can only get one of my three seizure meds because the other two have to be monitored and so I go through a lot of seizures a day.<br><br>[Researcher: How has your access to harm reduction services changed since COVID-19?] I've known them for an awful long time. I've saved a few lives given the fact that I had the Narcan to people that overdose, people that don't know how to use the drug, like heroin, and overdone it and almost died because of it. Yeah I still go [to get supplies from harm reduction services], but they ain't been coming out here an awful lot.<br><br>[Researcher: What do you think about wearing a face mask?] I can't breathe in it, because of my asthma.	Disability is … a handicap. Yeah it is. Because now I have this…I got this tingling, my feet swell up, I can't walk, and it gets you tired. I can't stand for long period of time because you get weak. And my job was being a mechanic so I'm stepping, stooping, bending, climbing. I couldn't perform so I would feel dizzy and fall and that's bad for the company then so I can't work. The staff here [at the hotel] they do the right thing for you. They help you in whatever way they're allowed to. I mean they're not nurses. But if you need help with anything like washing clothes, or when you move they help you move. It's very good. They're doing Hell of a job.<br><br>[Researcher: So I know you haven't been able to access some of the resources our group is trying to provide, but which things do you need access to?] I could use a social worker, and I really need help with my medications. If I did get the virus, that would be scary. I've tried…. Transportation is a huge issue for me. I wasn't able to make it to my doctor's appointment.
Sweeps	[Researcher: Where have the sweeps been?] Um [] Stockton Blvd yesterday. [Researcher: Can you describe what that's like?] For me, for when it happened to me, they broke my arm, they took my [breathing machine] away, and they broke my arm. [Researcher: "I'm so sorry. What was that experience like when they took your property and your breathing machine?"] It wasn't labeled, I guess, they didn't think it was important.	[Researcher: Have you been affected by police sweeps recently?] The sweeps as far as Sacramento City PD - the chief of police said that they aren't operating sweeps, but they are operating sweeps. They say they weren't, but every morning they're waking up homeless at 6 and 7 o clock in the morning and if you're not up they're giving you a citation. Some of the homeless bring it on themselves. You can't trash the sidewalk, you can't just throw trash everywhere and they do that. City Hall was being really cool but when some of the homeless decided that they would write the windows, they didn't like that so they started increasing. We

	[Researcher: Please describe if there have been any police sweeps recently and how they have affected you.] They're still harassing us, they're still being…I refuse to use the word 'cause it's really not worth it. Because they're only doing their jobs. I understand they're doing their jobs, for the most part, but I seriously think that they need more…human lessons on how to interact. For example, um, when that little boy Zion came up missing on Monday of last week? That night there was a police officer up above me on the bike trail who was on the PA system calling for the kid as he's cussing at the kid. I mean he's literally cussing this kid out! I know if I were a 5-year-old kid I wouldn't want to go towards that. And I had to listen to that until 6 o'clock in the morning when that cop went off duty. And I know that if I were that cop I'd be feelin' like a complete ass now because a couple hours after that happened, they found the kid dead. So basically he was cussing out a dead kid.<br><br>As long as we move we are alright. They do that all the time, on a regular basis. When they say homeless outreach, how they are outreaching is to take [our] property, that's the biggest thing they do.	made the paper one time because my wife gave an interview to Sac PD and after that - they didn't stop the sweeps - they'd just pull through and tell you to wake up and move on, but they didn't really make sure you got up anymore. The sweeps are a lot of trouble, especially with the [inaudible]. At first if you don't wake up they start going through your stuff and next thing you know you're going to jail.<br><br>[Researcher: Prior to the hotel, did you face any police sweeps where you were staying?] Yes – I got locked up for trespassing a couple times. I was on someone's land and I didn't know that. They called the cops on me and locked me up for second degree trespassing. [Researcher: No warning?] None. They just pulled up on me and cut my tent to shreds.
Barriers to Housing	[Researcher: What services do you still need?] I was on the housing list and I'm pretty much on hold now. Well, 'cause the housing where I was waiting on pretty much put everything on hold. Just through losing of the services. And um they pretty much set everything down because of the COVID-19.<br><br>[Researcher: In your life right now, what is your biggest fear?] Being out here any longer, I guess. I'm old. Getting older. It's a lot harder to survive out here. Um, what's my biggest fear? I think that's like a real fear. I mean you talk about a daily fear. I mean, being gay, being on the streets, is pretty dangerous. Yeah. Yeah, you know, when you're talking about all of this stuff, we have in the homeless community, we have quite a few transgender people, and because of that, they're kinda hidden away, and it's been really hard for them to get services because they have to actually come out and say "transgender."<br><br>[Researcher: You mentioned you were still waiting for a link to hotel housing; however, it was very important that your family stays together. Can you elaborate on that?] Well my 18-year-old son has [motions to indicate mental disability] so it's important that we maintain close contact because we are immediate family. Um, and like, so we are a family; we have three animals and just have to stay together. It's important for us to stay together. [Researcher: Any you were saying that representatives come out here?] They were. But they didn't find any housing that was suitable for our needs. Um, at first we were going to be in agreeance to, like, my son and I be in a room and him [her husband] take one of the dogs and I take two of the dogs. Just to make it work. But they also said that we would have to stay inside during the day which means that he and I wouldn't be able to work. And that didn't suit our needs, cause, so, cause we have a need to take care of	[Researcher: What are your plans for after staying in the hotel?] I don't have them. I mean my option is that I end up back in the woods, or last time I talked to my navigator, she said we could try to reapply to get me into another hotel if they're still doing it, under still being sick. See, I get told by the navigators that I'm a hard person to place into things cuz like the other day, last Friday when I was there, they were like well if you were HIV positive, we could get you in on a medical, if you tried to commit suicide, we could get you in on a psychiatric, if you were using drugs, we could get you in to a drug program, but you don't have any of those, so we don't know what to do with you.<br><br>[Researcher: What are your plans for after staying in the hotel?] I don't know how long this thing will last, I think it'll last for a while. I don't know. I'm hoping the housing council's gonna come around. But, me and my wife were talking about it. I think we're just gonna get a vehicle and I'm gonna try to go back to home health care, but I don't know how that's gonna work really. Maybe not have to wait for a government program to help us get into housing, it'd be nice, but we may have to do it ourselves. Stay here and not have to pay rent and we can save money. I just don't wanna go from here back to the streets. That would be a huge step back. Being on the street you get caught up in the homeless lifestyle and then it's hard to cross back over. That's the hard part.<br><br>[Researcher: Can you recap how you got to this housing program and what were some of the difficulties with that?] What was difficult was trying to find, trying to be able to do it. Most people when they're homeless, they got wagons, they got dogs - my wagon was four feet tall! You can only bring a limited amount of baggage with you, so that made it tough, that made it real hard. At the end, it just

	ourselves. I don't really like to rely on any government programs or anything if I don't have to…and other than that we didn't find any housing that fit our family unit. And it's important to me, it's the priority for me.<br><br>[Researcher: Please describe if you are aware of hotel rooms becoming available and what you think of this process.] Umm. I am waiting for one that is suitable for me. I am like a doctor I've got lots of patience. [Laughter]. Usually pray. I think they need more case by case. Open to families like that. SO that way like families like ours aren't broken up. Other than that? More animal friendly because I know other people who have…3 or more animals.<br><br>[Researcher: Please describe if you are aware of hotel rooms becoming available and what you think of this process.] They're not moving along fast enough. Because I was in housing before through Sacramento Steps Forward. And I just met with the house manager over a month ago. He dragged me out by my neck, my collar, [inaudible] dragged me out and locked the gate. I've been back there. I haven't been able to get in touch with Sacramento Steps Forward. Finally got in touch with this [inaudible] the other day, and told him I want a hotel, I'm going to go along with it. I want to get my life back together. I've been clean and sober long enough now. He tells me [inaudible]. I want out of the streets before I can get to [inaudible]. Leave me in the streets and there's going to be another million dollar hospital bill. I'm in a wheelchair now, but for the first five days it was 1.1 million dollars. It would be cheaper and better for all of us around to put me in a hotel room. It wouldn't be a million dollars.	took us leaving our tent and some blankets and pillows. We had to rush down to our storage and then we had to shoo all the way over to a church to meet them. I wasn't even sure if we were gonna be there in time. [Researcher: How much time would you say they gave you between when you would contact them and they would leave?] Usually the navigator would give you like a one hour notice. So we had to go down to the storage and then we had to hightail it over to 17th and L, so that was probably twenty blocks at least, probably a little more than that. So they were there, they were waiting, they waited almost an hour. They were calling my wife on her cell phone, but her cell phone died and there was no way to charge it. But we were probably within 5 blocks so they waited and they brought us out here. [Researcher: What determined the location that they would pick you up at? Did they tell you that was where they were going to meet you? Beforehand?] They gave us an hour notice and we couldn't do it because my wife googled it and said it was a thirty minute walk. And I was telling her no, it's like thirty minutes, like me and you going for a walk. But it's a lot different when you're carrying backpacks and fifty pounds of dog food and everything you own to put in storage. Of course we walked quicker from the storage to St Johns because all we had was two dogs leading us but they gotta go the bathroom and smell something and stop. It was alright. [Researcher: Before that did they give you any heads up where you were supposed to be to meet them?] First time we tried to do it, we couldn't do it, I told my wife we couldn't do it. The time frame, you can't make it because you don't have a car, we're on foot. By the time we paid for storage, I had to go back and get all our stuff. When we're halfway there, they waited at the church again, but we didn't show up, but they didn't give us enough time. It's not like I can throw everything in a vehicle and drive down in ten minutes.
Housing Access	[Researcher: In your life right now, what is your most important need?] That's to get myself and my mind together, and get up outta here. I'm not in a hole and um I wanna make sure that I um, hey, I'm good. Not that I'm crazy but I'm more than that.<br><br>These [unhoused] people are trying to find a place to live. They can't have 6 feet. You know, maybe they might try, or maybe they might even want to, but they can't because maybe 6 feet is out of the camp. They have no place to sleep. So it was really difficult for homeless people and low income people. Like for example, a whole family that lives in one bedroom. It just made it impossible for low income, for homeless people to really believe at first that there was really a pandemic. And then when they did believe it, they didn't have the resources to protect themselves.<br><br>[Researcher: Please describe how you identify in terms of your housing status.] I'm homeless. I got nothing. What you see here is something that I've gathered up due to a lot of people giving stuff up and go from there. Some of it's in good shape, some of it's not. You do what you can.	[Researcher: What do you like about the hotel situation?] That we're indoors. That we have meals. That we have cable. We have showers. We have bathrooms. It's safe, we're safe.<br><br>[Researcher: What would you change about the hotel situation?] Nothing. I'd like to stay longer, but nothing other than that.<br><br>[Researcher: What was your overall opinion of the process getting into a hotel?] It was kinda hard but it was actually kinda easy. I mean yes they have a lot of different ways of how they have to do it. And they have to do an intake and everything but it was easy to get into one I would say. Yeah it was actually worth the wait.<br><br>[Researcher: And what do you like about the hotel situation and what do you not like about the hotel situation?] Well they give you food um they help you out with the what you need up to a certain point but other than that it's been quiet. You don't get harassed like you would on the streets. You want it you don't get asked um if you have any drugs for sale or if you guys are looking for drugs. Now I

	[Researcher: Please describe how you identify in terms of your housing status.] Um, displaced member among society. I’m not homeless. [Researcher: And why?] Homeless is a harsh word, very harsh. When a person says your homeless, there’s a [inaudible] many shapes or forms. Homeless are cast aside as [inaudible], no good, don’t wanna waste our time with them when they can’t do this, can’t do that. They very stereotype homeless.<br><br>[Researcher: How do you identify in terms of your housing status, are you OK with the term homeless or do you use other terms or what’s appropriate to you?] Most people are never gonna be OK with being homeless…vast majority of homeless people they want out. They want an apartment. They want more.<br><br>[Researcher: Please describe if you are aware of hotel rooms becoming available and what you think of this process.] Depends on how long are they for and are we right back on the streets afterwards.<br><br>[Researcher: Please describe if you are aware of hotel rooms becoming available and what you think of this process.] Oh that is like the lady with the kitty cat? She’s in a hotel for like a month? I think it’s good. Well for the older people who have they immune system who can’t be out here, yea. Every now and then, I would like to take a bath. It’s good. I mean if you wanna go, you go. If not, you stay. So you have a choice. So that’s good. That’s good what they doin.<br><br>[Researcher: In your life right now, what is your most important need?] I say now the way things are, my most important need is getting some better housing. Like I said I can’t do that, I got no income, da-da-duh, so it’s… logically no way to even think like that. [Researcher: And do you have any more thoughts on COVID or our situation that you wanted to share? No, it’s been like just a joke…in terms of housing programs that have been offered, they do have a lot of restrictions, you know, they have rules on how you can only be out certain times of the day. That’s not helping me cuz all the money I make, I make by I canvasing houses. In the daytime, but still, you know, don’t tell me I got go all day and then can’t go to Home Depot at 6 and do what I need to do. Go talk to people, get work, you know. So the majority of the programs just won’t work for me.<br><br>[Researcher: In your life right now, what is your most important need?] Safety and stability.<br><br>[Researcher: In your life right now, what is your most important need?]<br>Housing, housing, housing.	don't do drugs personally, but you know it's some if it's that person's profession to do drugs well it's on them it’s not on me.<br><br>[Researcher: In your life right now, what is your most important need?] I been tryin' to correct my credit I had good credit back in 2001 and 2 and 3. Then uh, like I said I- I'm gonna look for a place and by then I should have enough money and uh if I can stay here enough time, have enough to put down a deposit and rent. And I'll be fine from that point on I just need that extra money [from Social Security] to get in the place, you know what I'm sayin'.<br><br>[Researcher: In your life right now, what is your biggest fear?] Oh yeah, the biggest fear is that I hope that when I come out of here I do have a place to go so I feel safe. When I don’t have a place to go I don’t feel safe. It’s gonna be hard. But I’m thinking by the time, the time comes, hopefully it’s all together. Hopefully. But living out here without a place to go, it’s the worst.<br><br>[Researcher: In your life right now, what is your most important need?] Housing. My housing and some place where I can be more – be yourself. It’s not here, it’s okay, it’s not bad, but it’s just temporary. I want some place where I’m going to be most of the time so I can plan. You can’t plan here if you don’t know what’s your next move. Being in some place where you can have a place to stay, say 2 years, that’s plenty of time to plan your life. Three months you might if you’re working hard and making some money. But in two years I hope to find a job, get more, get a little better, get a little bit more cash, depending my health-wise. Where I can move and you know, get a car or something, move to the doctor, do that and just feel more human instead of just being, like, you know having nowhere to go. That’s it. Other than that, I’m still not suicidal.<br><br>[Researcher: In your life right now, what is your most important need?] Housing. Yeah pretty much that’s it. [Researcher: Well I’m glad they’re able to bring out some people tomorrow hopefully.] That’s what we’re hearing so I’m hopeful. Well we heard that before and it didn’t happen so. [Researcher: At the hotel?] It was a different group of people, it wasn’t the housing people. I know they’re there, I know they’re coming, just tell us when they’re here.<br><br>[Researcher: Which resources provided to the unhoused community would be the most important to you moving forward?] Having a roof over my head is the most important thing. I lived in a tent for 5 years and had nothing and no one, just myself.
