# Supplementary material for: COVID-19 response and the unhoused communities in Sacramento: a mixed methods study with policy implications
Source: BMC Public Health. 2025 Nov 18;25:4012. doi: 10.1186/s12889-025-24515-0 (PMC12625094; doi:10.1186/s12889-025-24515-0)
Supplement: Supplementary file 8 — Additional file 8. Summary of representative quotes in the main text by theme [file 12889_2025_24515_MOESM8_ESM.pdf]

Additional file 8: Summary of representative quotes in the main text by theme

Access to Resources	Social Connection as Empowerment	Exacerbation of Pre-Existing Conditions	Impact of Systems & Policy on Safety
"I'm homeless. I got nothing. What you see here is something that I've gathered up due to a lot of people giving stuff up and go from there. Some of it's in good shape, some of it's not. You do what you can."<br><br>*Information*<br>*"It's hard to get any information. I've got more information since we've been here [at the hotel] and I've watched the news. The homeless can't just watch the news. Some have a radio, but most don't."*<br><br>*"I got a text from uh, a lady friend, she did a chain text, and she said, 'Hold your breath 10 seconds in the morning, and if you don't start coughing real bad after 10 seconds of holding your breath in this area, then you don't have it.' And I been doin' that ever since I read that."*<br><br>*"Now [if] you don't have a driver's license, you need an ID…you need [your] birth certificate…then it just becomes hard…you have no access to them…the office is not open."*<br><br>*Sanitation*<br>*"And now it's finally caught up with us. And we were dirty, we were not being clean enough. C'mon that's what this pandemic is all about. It's clean-li-ness. Homeless people can't be washing their hands every 20 minutes. It's impossible."*<br><br>*"You have all these people downtown, homeless with no restroom. And we kept hearing that they were gonna put washing stations in the park across the street – which they did – but they didn't maintain them. We were just kind of lost down there."*	*"Whatever can happen to you can happen to me. If I'm sick [with the virus] and I don't take care of myself, you can get sick [through transmission of the virus]. So, it's like people have to love themselves."*<br><br>*"A lot of people look down on being homeless. A lot of people look at us like the scum of the earth sometimes. I have met some of the greatest people – who are homeless. Some of the people with houses, not so beautiful. I do say money is the root to all evil. So, being broke has never been my problem… When they judge a book by its cover they might miss a great story."*<br><br>*Sanitation as a Community Action*<br>*"We are considered the lost, the not worthy of anything in life...we are treated as garbage. I have physical conditions that make it hard for me to even be able to use the restroom with some form of dignity and then feel like there is some kind of sanitation."*<br><br>*"For a while, I got known to where, you know, I've had park rangers give me trash bags 'cause they'd see me picking up trash, and now if every homeless person did that, we wouldn't get fines...They'd see that eh – at least we're, you know, doing a good job keeping everything clean."*<br><br>*Trust with Providers*<br>*"What was most important – what hit my heart? The [students] would come around to the homeless people, on their own time, and offer hand sanitizer and ask you what's wrong with you, if you need any help: Do you need any suggestions on where to go or what to do? That has probably meant the most."*<br><br>*"I don't really talk to them [unfamiliar outreach workers]. I talk to you guys."*	*"It's not just the epidemic, it's my health. So I'm not trying to blame corona[virus], I blame my health and yeah you blame it on yourself for not taking care of it. … You have to take your medication before your food. But if you dunno what food you're gonna eat, how are you gonna take your medicine? So I wouldn't take my medicine then…"*<br><br>*Chronic Health Conditions*<br>*"I could use a social worker, and I really need help with my medications. If I did get the virus, that would be scary. … Transportation is a huge issue for me. I wasn't able to make it to my doctor's appointment."*<br><br>*"I actually had to turn down my insulin because of the fact that I have no reason or no way to keep it cold no more 'cause I [now] have no access to the ice machine."*<br><br>*"I'm epileptic; and I can only get one of my three seizure meds because the other two have to be monitored, and so I go through a lot of seizures a day."*	*Sweeps*<br>*"As long as we move, we are alright. They do that all the time, on a regular basis. When they say homeless outreach, how they are outreaching is to take [our] property – that's the biggest thing they do… When we are moved around like that it takes our energies and focus away from doing things that can be more preventative from us being out here. You know what I mean? Finding jobs or places we can be. When they are shuffling us around like that, all that does is exhaust us, tear our health down, and kill us off, little by little, really. Or incriminate us by taking us to jail."*<br><br>*Barriers to Housing*<br>*"Well, my 18-year-old son has [motions to indicate mental disability], so it's important that we maintain close contact because we are immediate family. But they also said that we would have to stay inside during the day, which means that he [her husband] and I wouldn't be able to work…and we didn't find any housing that fit our family unit. And it's important to me; it's the priority for me."*<br><br>*"I get told by the navigators that I'm a hard person to place. The other day, last Friday when I was there, they were like well if you were HIV positive, we could get you in on a medical, if you tried to commit suicide, we could get you in on a psychiatric, if you were using drugs, we could get you in to a drug program, but you don't have any of those, so we don't know what to do with you."*<br><br>*"I got two pets and they've saved my life many times…'cause they're federal registered service dogs. Through myself training them, and plus just the knowledge of the dogs themselves, they've- they've stopped a car from running me over. They've found me when I got lost, they help me get to my camp sites when I get lost."*<br><br>*"And there [were] times where my dog ate and that was good enough for me."*<br><br>*Housing Access*<br>*"I say now the way things are, my most important need is getting some better housing. Like I said, I can't do that, I got no income, da-da-duh, so it's… logically no way to even think like that."*<br><br>*"That's to get myself and my mind together, and get up outta here. I'm not in a hole and um I wanna make sure that I um, hey, I'm good. Not that I'm crazy but I'm more than that."*<br><br>*"I been tryin' to correct my credit. I had good credit back in 2001 and 2 and 3. Then uh, like I said, I- I'm gonna look for a place and by then I should have enough money, and, uh, if I can stay here enough time, have enough to put down a deposit and rent. And I'll be fine from that point on. I just need that extra money [from Social Security] to get in the place, you know what I'm sayin'."*<br><br>*"We're indoors … We have showers. We have bathrooms. It's safe; we're safe."*<br><br>*"[I need a] roof over my head – my own roof. I can't stay with people. That's what my navigator is trying to do, find me my own. I think God's giving me a second chance."*<br><br>*"There needs to be classes to make it more successful for a homeless person to go back to society and be regular. I don't think that they realize – I've seen it, I've seen it many times – and then they lose their homes."*

Subthemes noted in gray.
