# Supplementary material for: COVID-19 response and the unhoused communities in Sacramento: a mixed methods study with policy implications
Source: BMC Public Health. 2025 Nov 18;25:4012. doi: 10.1186/s12889-025-24515-0 (PMC12625094; doi:10.1186/s12889-025-24515-0)
Supplement: Supplementary file 9 — Additional file 9. Number of interview transcript analyses involving the use of each code [file 12889_2025_24515_MOESM9_ESM.pdf]

Additional file 9: Number of interview transcript analyses involving the use of each code[1]

| **Code Category: Code**[2] | **Outside PEH** *n* = 19 | **Hotel PEH** *n* = 16 | **All PEH** *n* = 35 |
|---|---|---|---|
| | | | |
| any: ACCESS | **17 (89%)**[3] | **15 (94%)** | **32 (91%)** |
| any: CAUSE | 14 (74%) | 9 (56%) | 23 (66%) |
| any: CHANGE | **16 (84%)** | **13 (81%)** | **29 (83%)** |
| any: COPING | 11 (58%) | 8 (50%) | 19 (54%) |
| any: FEAR | **18 (95%)** | **16 (100%)** | **34 (97%)** |
| any: FUTURE | 13 (68%) | **14 (88%)** | **27 (77%)** |
| any: HUMANITY | **15 (79%)** | 11 (69%) | 26 (74%) |
| any: IDENTITY | **19 (100%)** | **16 (100%)** | **35 (100%)** |
| any: INDIFF | 11 (58%) | 9 (56%) | 20 (57%) |
| any: INFO | **19 (100%)** | **15 (94%)** | **34 (97%)** |
| any: NEED | **19 (100%)** | **15 (94%)** | **34 (97%)** |
| any: PRIORITY | **17 (89%)** | **15 (94%)** | **32 (91%)** |
| any: RESPECT | 11 (58%) | 7 (44%) | 18 (51%) |
| any: SLICE OF LIFE | 5 (26%) | 9 (56%) | 14 (40%) |
| any: SOLUTION | 13 (68%) | **13 (81%)** | 26 (74%) |
| any: SPIRITUALITY | 9 (47%) | 6 (38%) | 15 (43%) |
| any: STRESS | 11 (58%) | 10 (63%) | 21 (60%) |

| | | | |
|---|---|---|---|
| any: TEAM | 12 (63%) | 7 (44%) | 19 (54%) |
| any: TECHNOLOGY | 12 (63%) | **14 (88%)** | 26 (74%) |
| any: TRANSPARENCY | 12 (63%) | 6 (38%) | 18 (51%) |
| any: TRUST | **17 (89%)** | **13 (81%)** | **30 (86%)** |
| any: UNCERTAINTY | 9 (47%) | 11 (69%) | 20 (57%) |
| | | | |
| BASIC NEEDS: CLOTHING | 4 (21%) | 2 (13%) | 6 (17%) |
| BASIC NEEDS: FOOD | **19 (100%)** | **15 (94%)** | **34 (97%)** |
| BASIC NEEDS: ID CARD | 3 (16%) | 2 (13%) | 5 (14%) |
| BASIC NEEDS: MONEY | 12 (63%) | 11 (69%) | 23 (66%) |
| BASIC NEEDS: NETWORK | **19 (100%)** | **16 (100%)** | **35 (100%)** |
| BASIC NEEDS: PET | **15 (79%)** | **12 (75%)** | **27 (77%)** |
| BASIC NEEDS: SAFETY | 7 (37%) | 7 (44%) | 14 (40%) |
| BASIC NEEDS: SANITATION | **18 (95%)** | **15 (94%)** | **33 (94%)** |
| BASIC NEEDS: SHELTER | 2 (11%) | 7 (44%) | 9 (26%) |
| BASIC NEEDS: TRANSPO | 13 (68%) | 10 (63%) | 23 (66%) |
| BASIC NEEDS: WATER | **17 (89%)** | 9 (56%) | 26 (74%) |
| | | | |
| HEALTH: BENEFIT | 8 (42%) | 2 (13%) | 10 (29%) |
| HEALTH: COMMUNITY-RISK | **16 (84%)** | 11 (69%) | **27 (77%)** |
| HEALTH: COVID | **19 (100%)** | **16 (100%)** | **35 (100%)** |
| HEALTH: DISABILITY | 8 (42%) | 4 (25%) | 12 (34%) |

| | | | |
|---|---|---|---|
| HEALTH: HARM | 8 (42%) | 7 (44%) | 15 (43%) |
| HEALTH: HARM REDUCTION | 11 (58%) | 7 (44%) | 18 (51%) |
| HEALTH: HEALTHCARE | **19 (100%)** | **16 (100%)** | **35 (100%)** |
| HEALTH: IMMUNITY | 5 (26%) | 3 (19%) | 8 (23%) |
| HEALTH: MANAGEMENT | 12 (63%) | 7 (44%) | 19 (54%) |
| HEALTH: MENTAL | 10 (53%) | 8 (50%) | 18 (51%) |
| HEALTH: OTHER | 4 (21%) | 5 (31%) | 9 (26%) |
| HEALTH: PREVENT | **16 (84%)** | **16 (100%)** | **32 (91%)** |
| HEALTH: REPRO | 1 (5%) | 0 (0%) | 1 (3%) |
| HEALTH: SELF-RISK | **17 (89%)** | **12 (75%)** | **29 (83%)** |
| HEALTH: SURGERY | 1 (5%) | 0 (0%) | 1 (3%) |
| HEALTH: TESTING | **18 (95%)** | **15 (94%)** | **33 (94%)** |
| HEALTH: TRAUMA | 11 (58%) | 9 (56%) | 20 (57%) |
| | | | |
| HOUSING: DISPLACEMENT | 13 (68%) | **12 (75%)** | 25 (71%) |
| HOUSING: HOUSE | **16 (84%)** | **15 (94%)** | **31 (89%)** |
| HOUSING: STABILITY | 10 (53%) | **14 (88%)** | 24 (69%) |
| | | | |
| POWER: EXTERNAL | **18 (95%)** | **14 (88%)** | **32 (91%)** |
| POWER: INPUT | 3 (16%) | 8 (50%) | 11 (31%) |
| POWER: SELF | **17 (89%)** | **12 (75%)** | **29 (83%)** |
| | | | |

| | | | |
|---|---|---|---|
| PUBLIC SERVICES: CRIME | 1 (5%) | 4 (25%) | 5 (14%) |
| PUBLIC SERVICES: GOVERNMENT | 9 (47%) | 8 (50%) | 17 (49%) |
| PUBLIC SERVICES: INCARCERATION | 3 (16%) | 3 (19%) | 6 (17%) |
| PUBLIC SERVICES: LEGAL | 3 (16%) | 3 (19%) | 6 (17%) |
| PUBLIC SERVICES: POLICE | **18 (95%)** | **13 (81%)** | **31 (89%)** |
| | | | |
| SUBSTANCE: METH | 0 (0%) | 3 (19%) | 3 (9%) |
| SUBSTANCE: OTHERSUB | 4 (21%) | 5 (31%) | 9 (26%) |
| | | | |
| QUOTE: GREAT | 10 (53%) | 7 (44%) | 17 (49%) |
| QUOTE: REC | 8 (42%) | 7 (44%) | 15 (43%) |
| QUOTE: UNIQUE | 8 (42%) | 8 (50%) | 16 (46%) |
| | | | |

[1] An interview transcript analysis was included in the count if the code of interest had been utilized at least once after completion of final coding.

[2] Refer to Additional file 5 for descriptions of each code.

[3] All data representing percentages greater than or equal to 75 are highlighted in bold to recognize common topics that played a larger role in informing subthemes, themes, and representative quote selection to unpack the heterogenous experiences of all PEH.
