# Supplementary material for: COVID-19 response and the unhoused communities in Sacramento: a mixed methods study with policy implications
Source: BMC Public Health. 2025 Nov 18;25:4012. doi: 10.1186/s12889-025-24515-0 (PMC12625094; doi:10.1186/s12889-025-24515-0)
Supplement: Supplementary file 10 — Additional file 10. Interview responses to the question: What is your most important need now? [file 12889_2025_24515_MOESM10_ESM.pdf]

Additional file 10: Interview responses to the question: *What is your most important need now?*

**Response Category**	**Outside PEH** *n* = 19	**Hotel PEH** *n* = 16	**All PEH** *n* = 35
housing	5 (26%)	9 (56%)	14 (40%)
idea[1]	4 (21%)	3 (19%)	7 (20%)
family[2]	6 (32%)	1 (6%)	7 (20%)
money	1 (5%)	2 (13%)	3 (9%)
health[3]	1 (5%)	1 (6%)	2 (6%)
item[4]	2 (11%)	0 (0%)	2 (6%)

[1] Defined as, in relation to an ideal feeling (ex. living life to the fullest; peace, love, and harmony)

[2] Defined as, in relation to social connections (ex. relatives; loved ones; companion animals)

[3] Defined as, if the specific stated goal is to stay healthy

[4] Defined as, in relation to a specific resource (ex. blankets; handwashing station)
